# Vacuum-Deposited Wide-Bandgap Perovskite for All-Perovskite Tandem Solar Cells

**DOI:** 10.1021/acsenergylett.3c00564

**Published:** 2023-05-24

**Authors:** Yu-Hsien Chiang, Kyle Frohna, Hayden Salway, Anna Abfalterer, Linfeng Pan, Bart Roose, Miguel Anaya, Samuel D. Stranks

**Affiliations:** † Cavendish Laboratory, Department of Physics, University of Cambridge, JJ Thomson Avenue, Cambridge CB3 0HE, United Kingdom; ‡ Department of Chemical Engineering & Biotechnology, University of Cambridge, Philippa Fawcett Drive, Cambridge CB3 0AS, United Kingdom

## Abstract

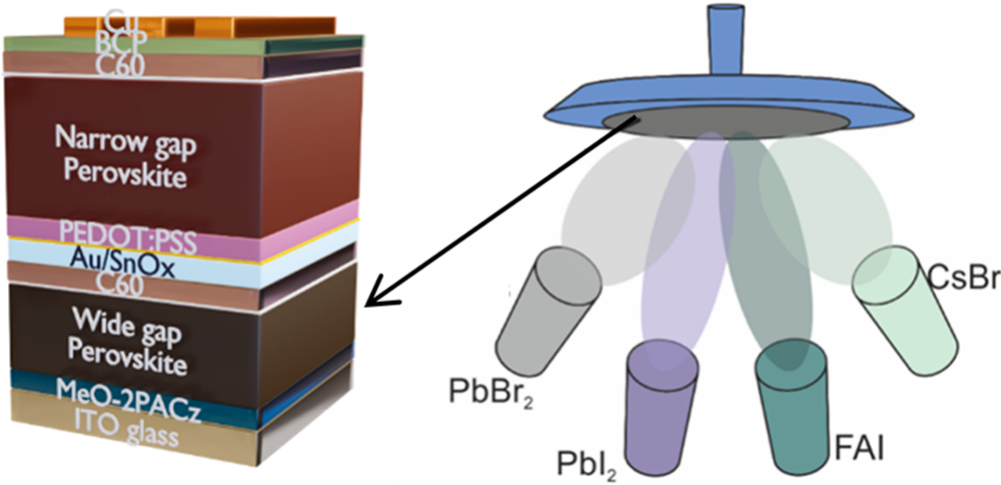

All-perovskite tandem solar cells beckon as lower cost alternatives to conventional single-junction cells. Solution processing has enabled rapid optimization of perovskite solar technologies, but new deposition routes will enable modularity and scalability, facilitating technology adoption. Here, we utilize 4-source vacuum deposition to deposit FA_0.7_Cs_0.3_Pb(I_*x*_Br_1–*x*_)_3_ perovskite, where the bandgap is changed through fine control over the halide content. We show how using MeO-2PACz as a hole-transporting material and passivating the perovskite with ethylenediammonium diiodide reduces nonradiative losses, resulting in efficiencies of 17.8% in solar cells based on vacuum-deposited perovskites with a bandgap of 1.76 eV. By similarly passivating a narrow-bandgap FA_0.75_Cs_0.25_Pb_0.5_Sn_0.5_I_3_ perovskite and combining it with a subcell of evaporated FA_0.7_Cs_0.3_Pb(I_0.64_Br_0.36_)_3_, we report a 2-terminal all-perovskite tandem solar cell with champion open circuit voltage and efficiency of 2.06 V and 24.1%, respectively. This dry deposition method enables high reproducibility, opening avenues for modular, scalable multijunction devices even in complex architectures.

Multijunction solar cells constitute the most practical way to achieve power conversion efficiencies (PCEs) beyond the radiative efficiency limits of single-junction solar cells. Multijunction technologies employ photoabsorbers with complementary bandgaps to collectively harvest a broader portion of the solar spectrum while minimizing thermalization losses upon hot-carrier relaxation. The highest performance of a solar cell reported to date is from a triple junction based on III–V semiconductors with a strain-balanced quantum well stack, achieving a PCE of 39.5%.^1^ However, their high cost due to complex fabrication processes that involve high temperatures limits their accessibility and versatility. These costs are historically limiting the use of III–V solar cells in terrestrial power applications and restrict their use to high-value applications such as powering satellites or space vehicles.

Halide perovskites are generating enormous excitement as thin-film absorbers for high-performance solar cells, showing a unique combination of features that include low-temperature processing and a resilience to electronic defects.^2^ With a compositionally tunable ABX_3_ crystal structure, where A = methylammonium (MA), formamidinium (FA), and/or Cs, B = Pb and/or Sn, and X = Cl, Br, I, the bandgap of 3D perovskites can be varied from 1.2 to 3.0 eV. This absorption tunability, combined with high absorption coefficients and charge carrier mobilities, makes these materials promising for both single- and multiple-junction thin-film solar cells.^3−6^ Indeed, the record PCE single-junction perovskite and all-perovskite tandem solar cells have reached PCEs to date of 25.7% and 27.4%,^7,8^ respectively, representing the most efficient emerging PV systems to date. These outstanding outcomes result from years of work from myriads of research groups mostly working with solution-processed approaches that allow rapid screening and optimization. However, solution approaches ultimately present limitations for manufacturing due to the use of toxic solvents and potential issues with dissolving underlying layers, the latter limiting the underlying materials and substrates.

Vacuum deposition processes show great promise to overcome barriers related to large-area coating, integration into flexible, lightweight substrates, and novel device patterns while ensuring high thickness control and conformal film uniformity, all with a solvent-free technique. To date, fully evaporated perovskite solar cells have achieved PCEs of 20.7% and 21.4% on a small active area (<0.2 cm^2^) by coevaporation and sequential evaporation, respectively,^9,10^ and a PCE of 18.1% over a larger area (21 cm^2^).^11^ While the community has concentrated most efforts on evaporating MAPbI_3_ perovskite solar cells,^9,11−13^ Li et al. and Wang et al. have reported MA-free perovskite solar cells with high thermal stability, though their perovskite fabrication processes are hybrid or sequential depositions.^14,15^ Our group and others have demonstrated that MA-free, mixed halide multisource evaporation systems are viable candidates to achieve thermally stable perovskite devices.^16−19^ Importantly, the dry nature of the technique represents an ideal approach to stack different perovskite films for tandem device architectures on a range of underlying contacts and substrates, an approach that opens avenues for a highly efficient yet low-cost thin-film, lightweight perovskite technology.

Nevertheless, only a handful of works have reported fully evaporated perovskites for their application in multijunction cells, with most of the examples focusing on deposition processes to combine perovskite and silicon subcells in a tandem fashion.^20−22^ As for all-perovskite tandem systems, Ávila et al. reported a vacuum-deposited MAPbI_3_-MAPbI_3_ perovskite solar cell with an outstanding *V*_OC_ of 2.3 eV and a PCE of 18%, demonstrating the potential of the technique to attain building blocks for tandem devices.^23^ However, perovskite bandgaps in this work were not optimized to minimize energy losses while maximizing current matching for AM1.5 illumination. Optical modeling suggests that a PCE >35% cell efficiency is potentially achievable in all-perovskite tandems under realistic conditions.^24−26^ This value is conditioned by the absorption spectrum of the rear subcell, as the narrowest perovskite bandgaps demonstrated so far are in the range between 1.20 and 1.30 eV based on alloyed Pb/Sn compositions. The bandgap of the optimum front (wide-bandgap) subcell for that constraint is between 1.70 and 1.80 eV, though little further loss is seen when the bandgap is lowered further to 1.65 eV when light coupling between layers is taken into account.^27^ One challenge in realizing these wide-bandgap perovskite materials is that they inevitably require mixed halide compositions, and they hence suffer from light-induced phase segregation, forming Br and I rich subdomains^28−31^ that reduce the open-circuit voltage (*V*_OC_).^32^ Gil-Escrig et al. reported a wide-bandgap (1.77 eV) perovskite with a composition of FA_0.61_Cs_0.39_Pb(I_0.70_Br_0.30_)_3_ displaying a *V*_OC_ of up to 1.21 V, the best to date for a vacuum-deposited system.^16^ Yet, this *V*_OC_ is still 240 mV below the radiative efficiency limit of 1.45 V for a 1.77 eV bandgap, indicating that there are still significant losses in the best evaporated, wide-bandgap perovskite solar cells. Interestingly, it has been recently shown that low radiative efficiency in bulk mixed halide perovskites and energy misalignment between the perovskite and contact layers are the main losses in wide-bandgap perovskite solar cells, and a device *V*_OC_ of over 1.33 V (for a 1.77 eV bandgap) is achievable even in the presence of halide segregation.^32,33^ These results overall show the complex compromise between perovskite phase stabilization and device stack optimization required to attain vacuum-deposited wide-bandgap perovskite solar cells relevant for tandem architectures.

In this work, we employ a 4-source coevaporation technique to systematically vary the bandgap of FA_0.7_Cs_0.3_Pb(I_*x*_Br_1–*x*_)_3_ films from 1.62 to 1.80 eV for their subsequent integration in an all-perovskite tandem device. We show how contact layer optimization using (2-(3,6-dimethoxy-9*H*-carbazol-9-yl)ethyl)phosphonic acid (MeO-2PACz) as the hole-transporting material (HTM) leads to PCEs of 20.7% for a 1.62 eV perovskite, which is among the highest PCEs reported for a vacuum-deposited perovskite system. The addition of higher Br fractions to blue-shift the absorption onset for wide-bandgap subcells introduces defects, as demonstrated by a reduction in the photoluminescence quantum efficiency (PLQE) and a higher Urbach energy, which is particularly exacerbated at 1.80 eV, where substantial phase segregation readily occurs. Here, we show that ethylenediammonium diiodide (EDAI_2_), recently proposed for solution-processed devices,^34^ is also an effective surface passivation agent for evaporated perovskites, resulting in PLQEs enhanced by an order of magnitude. Applying EDAI_2_ interface treatment yields devices with a *V*_OC_ of 1.26 V for a 1.76 eV bandgap, which is 190 mV below the radiative limit and represents the lowest *V*_OC_ loss reported in evaporated wide-bandgap perovskite systems so far. We make use of this evaporated wide-gap solar cell to demonstrate an MA-free 2-terminal tandem device with a PCE of 24.1%, the highest for an all-perovskite tandem solar cell where at least one of the subcells is evaporated. This result shows the potential of the scalable and industry-relevant evaporation technique for realizing efficient and modular all-perovskite tandem solar cells.

Defects at the perovskite-hole-transporting-layer interface are known to cause significant nonradiative losses in p-i-n devices,^34^ limiting their applicability for tandem architectures where high voltages are required. Al-Ashouri et al. reported that the nonradiative losses arising from the interface between a perovskite and the typically employed poly[bis(4-phenyl)(2,4,6-trimethylphenyl)amine] (PTAA) can be substantially reduced when replacing the latter by a self-assembled monolayer (SAM), MeO-2PAC or 2PACz, leading to a higher device *V*_OC_.^35,36^ In order to explore this effect and optimize MA-free evaporated systems, we fabricate devices with an architecture consisting of ITO/HTM/perovskite (500 nm)/C60 (25 nm)/BCP (8 nm)/Cu. We initially employ our recently reported 3-source evaporation protocol^19^ to deposit FA_0.7_Cs_0.3_Pb(I_0.9_Br_0.1_)_3_ absorbers (Figure 1a with no PbBr_2_) on different HTMs, namely 2PACz, PTAA, and MeO-2PAC, with the absorber exhibiting a bandgap of 1.62 eV extracted via the inflection point of an external quantum efficiency (EQE) measurement (Figure S1). Scanning electron microscopy (SEM, Figure S2) images do not show significant differences in surface morphology, suggesting that the perovskite growth is similar on these different organic layers. We observe reduced nonradiative recombination in the perovskite/MeO-2PACz structure, with the PLQE being factors of 3.3 and 5 times higher than those of a perovskite deposited on 2PACz and PTAA, respectively (Figure S3). Figure 1b shows that the MeO-2PACz-based devices display a substantially higher *V*_OC_ (1.11 V on average) and less *V*_OC_ variation between devices than PTAA, with the trend being consistent with higher PLQE^36^ and across different batches (Figure S4). The devices based on 2PACz show s-kinks in the current–voltage (*J*–*V*) curves, resulting in very low performance (Figure S5 and Table S1). This result is in quite stark contrast to the high performance achieved in solution-processed systems on 2-PACz, even though we employ the same deposition parameters for all HTMs.^35,36^ The reason for the consistently low performance, especially *V*_OC_, in the 2PACz devices is currently unclear, though suboptimal interfaces between the evaporated perovskite and 2PACz may play a role. We note that the reoptimization of different evaporated perovskite compositions and deposition parameters might yield improved performance on 2PACz. Our champion device reaches a PCE of 20.7% when the evaporated perovskite is deposited on MeO-2PACz (Figure 1c), which is the highest PCE for MA-free perovskite solar cells reported for multisource evaporation to date. Further, the device retains ∼93% of its initial performance after 120 h under 1 sun illumination at a fixed bias set to the maximum power point with no substrate temperature control during the measurement (Figure 1d). We find that this front interface optimization is critical to ensure maximized voltages for subsequent integration of the cells as building blocks in tandem devices.

**Figure 1:**
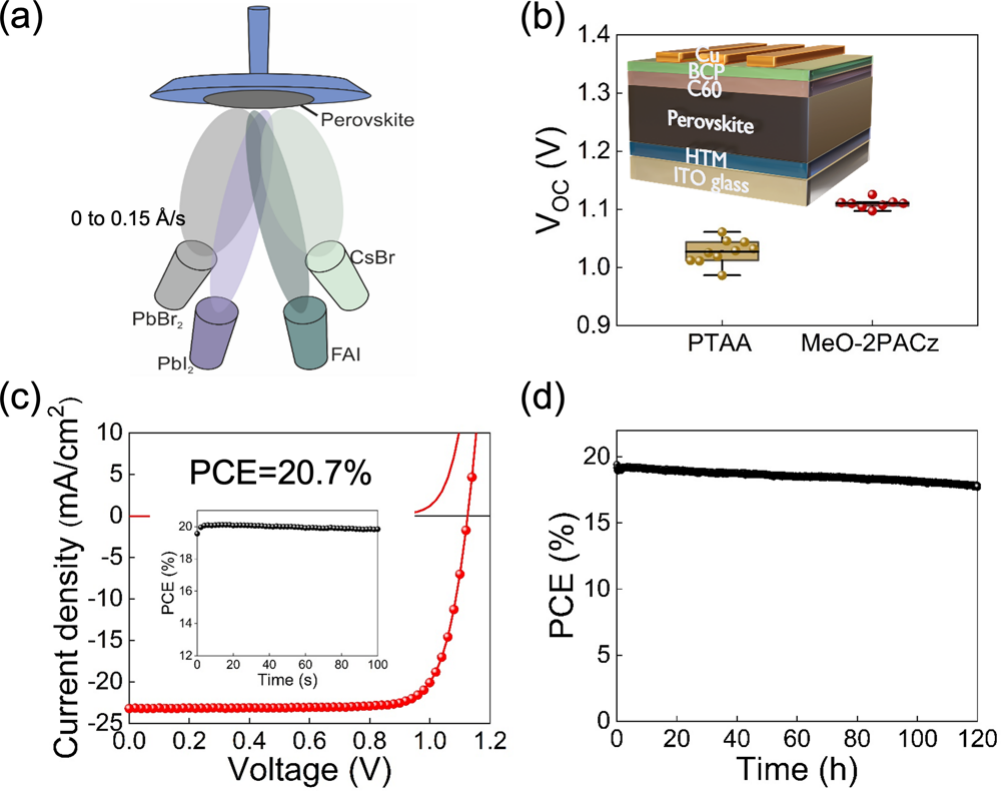
(a) Diagram of the thermal evaporation system where four sources (i.e., FAI, PbBr_2_, PbI_2_, and CsBr) are employed to deposit high-quality FA_0.7_Cs_0.3_Pb(I_*x*_Br_1–*x*_)_3_ thin films. FAI, PbI_2_, and CsBr evaporation rates are kept at 1, 0.6, and 0.1 Å/s, respectively, and the PbBr_2_:PbI_2_ deposition rate ratio was varied to tune the bandgap. (b) Effect of different HTMs (i.e., PTAA and MeO-2PACz) on the *V*_OC_ statistics of evaporated FA_0.7_Cs_0.3_Pb(I_0.9_Br_0.1_)_3_ perovskite solar cells (PbBr_2_ rate is 0). Inset: the device architecture employed. The box/whisker plot contains the 1.5 interquartile range, the median value, and data distribution from 8 devices. (c) *J*–*V* curve of the champion FA_0.7_Cs_0.3_Pb(I_0.9_Br_0.1_)_3_ perovskite solar cells with the inset showing a stabilized power output measurement. (d) Operational stability test of an encapsulated device at 0.94 V fixed bias under continuous 1 sun illumination.

To widen the bandgap of the perovskite for its use as a front subcell in a tandem device, we add PbBr_2_ as a fourth evaporation source to tune the bandgap by employing PbBr_2_:PbI_2_ rate ratios from 0.11 to 0.32. XRD patterns shown in Figure 2a confirm Br incorporation into the perovskite structure as the PbBr_2_ evaporation rate is increased, with the (011 if using cubic assignment, ∼14°) perovskite peak shifting to higher 2θ as a consequence of a smaller *d* spacing (see Figure S6 for full XRD patterns). Top-view SEM images (Figure S7) show perovskite grain sizes in the range of 100–300 nm for all compositions as well as what is likely the presence of PbI_2_ evidenced by bright clusters, consistent with our previous work and the PbI_2_ signal observed in the XRD patterns.^37^ In a previous report, we showed that the presence of PbI_2_ in excess in the vacuum-deposited perovskite enhances the optoelectronic properties and film stability on exposure to ambient conditions.^19^ We estimate the stoichiometry of the evaporated perovskite films using XRD across a range of control films to generate a calibration curve (Figure S8)^19^ and display the resulting chemical formulas in Table 1. We determine the corresponding bandgaps as the inflection point of the first derivative in the EQE spectrum (Figure S1), observing that the bandgap varies between 1.62 and 1.80 eV (Table 1), confirming the increase in bandgap upon additional Br incorporation. To understand this observation and obtain further insight into the chemical composition, we perform synchrotron-based nano-X-ray fluorescence (nXRF) measurements on our samples. The nanoprobe nature of the technique allows us to extract Br:Pb maps with a spatial resolution of ∼50 nm (Figure 2b). Evaporated perovskites with bandgaps between 1.62 and 1.77 eV show excellent halide spatial homogeneity, which is particularly striking when comparing to a standard solution-processed “triple-cation” FA_0.79_MA_0.16_Cs_0.05_Pb(I_0.83_Br_0.17_)_3_ perovskite film (bandgap of 1.62 eV), where we have found that the compositional heterogeneity is related to defects and carrier funneling.^2,38^ Nevertheless, the 1.80 eV bandgap evaporated film exhibits several areas with Br-rich clusters, suggesting a suboptimal intermixing of compounds in samples with the highest explored Br content, which is known to drastically hamper stability.^39^

**Figure 2:**
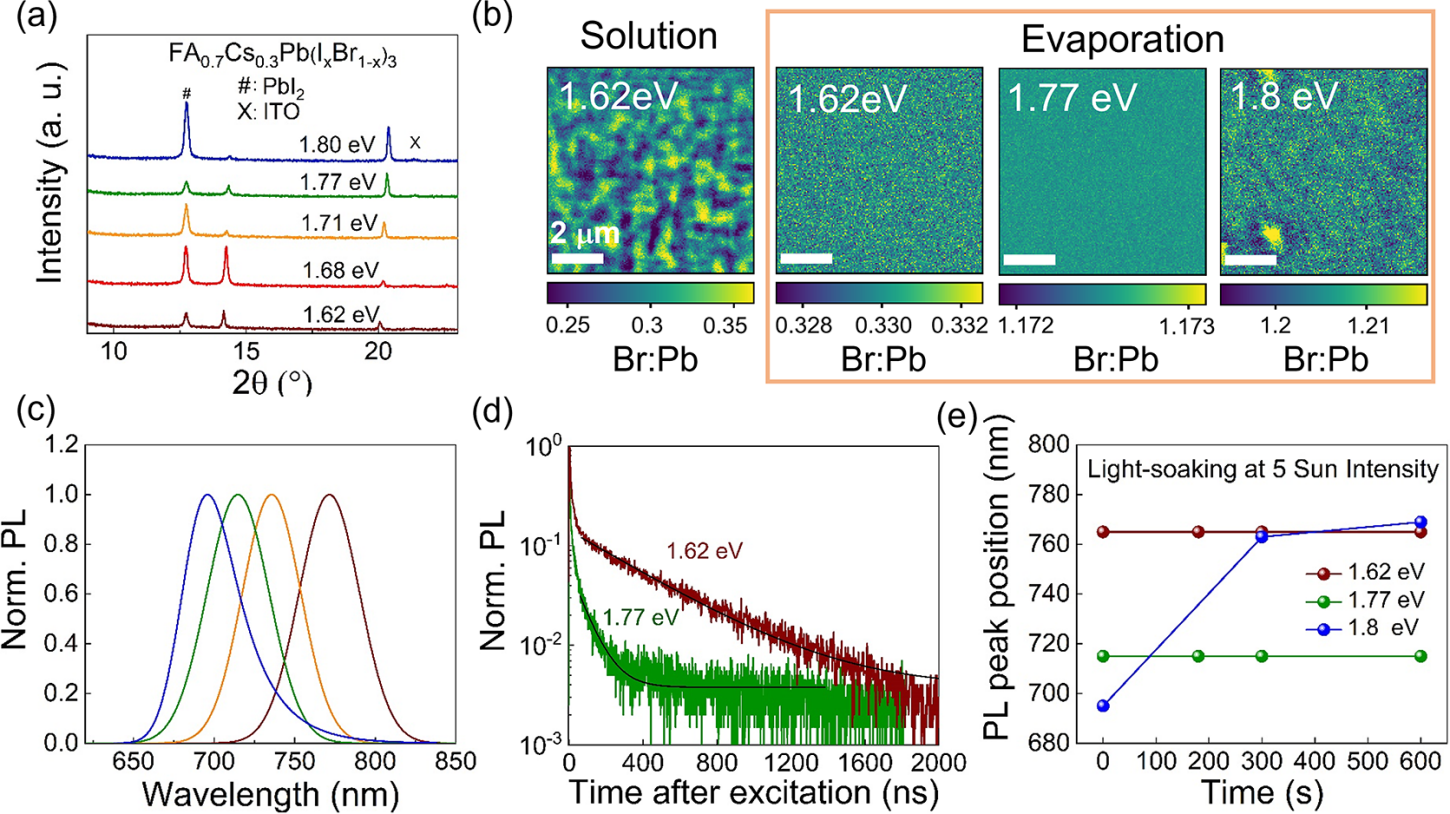
(a) XRD pattern of evaporated FA_0.7_Cs_0.3_Pb(I_*x*_Br_1–*x*_)_3_ thin films deposited on MeO-2PACz/ITO showing different bandgaps: 1.62 eV (dark red curve), 1.68 eV (red), 1.71 eV (orange), 1.77 eV (green), and 1.80 eV (blue). (b) Br:Pb obtained by nXRF mapping for a solution-processed triple-cation perovskite and evaporated perovskites with bandgaps of 1.62, 1.77, and 1.80 eV. Scale bars are 2 μm. (c) PL spectra of a series of evaporated FA_0.7_Cs_0.3_Pb(I_*x*_Br_1–*x*_)_3_ thin films deposited on MeO-2PACz/ITO showing different bandgaps: 1.62 eV (dark red curve), 1.71 eV (orange), 1.77 eV (green) and 1.80 eV (blue). (d) TRPL decays for 1.62 and 1.77 eV evaporated perovskite films on MeO-2PACz excited with a 450 nm laser at a fluence of 8.5 nJ/(cm^2^ pulse). Samples were excited, and light was collected from the top side. The black curves are fittings of the TRPL data (see Methods for details). (e) PL peak evolution over time for the 1.62, 1.77, and 1.80 eV evaporated perovskite films under continuous illumination at 5 sun (300 W/cm^2^) with a 520 nm laser.

**Table 1:** Champion PV Performance Metrics for Evaporated Perovskite Solar Cells with Different Bandgaps

bandgap (eV)	*V*_OC_ (V)	*J*_SC_ (mA/cm^2^)	FF (%)	PCE (%)	composition	PbBr_2_ rate (Å/s)
1.62	1.11	–23.0	78.7	20.0	FA_0.7_Cs_0.3_Pb(I_0.9_Br_0.1_)_3_	0
1.68	1.14	–19.8	78.5	17.6	FA_0.7_Cs_0.3_Pb(I_0.79_Br_0.21_)_3_	0.06
1.71	1.18	–19.1	78.4	17.7	FA_0.7_Cs_0.3_Pb(I_0.76_Br_0.24_)_3_	0.1
1.77	1.24	–18.5	69.2	15.9	FA_0.7_Cs_0.3_Pb(I_0.64_Br_0.36_)_3_	0.127
1.80	1.23	–17.4	72.9	15.6	FA_0.7_Cs_0.3_Pb(I_0.56_Br_0.44_)_3_	0.15

Steady-state photoluminescence (PL) measurements also reflect this variation (Figure 2c), with a clear tunability in the PL peak position from 1.62 to 1.80 eV. Evaluation of the charge carrier lifetime by time-resolved photoluminescence (TRPL) indicates lower optoelectronic quality in the wide-bandgap perovskites when compared with the 1.62 eV counterparts (Figure 2d). Both samples show a common quick decay in the first 40 ns attributed to quenching by the contacts. The subsequent PL decay of the 1.77 eV evaporated perovskite deposited on top of the MeO-2PACz/ITO contact shows faster (84 ns) monoexponential decay with respect to that of the control 1.62 eV sample (395 ns),^40,41^ where monoexponential decays are expected in experiments with such low carrier densities.^42^ We attribute the faster decay associated with Shockley–Read–Hall (SRH) recombination to an increase in the trap density when we replace fractions of I by Br in the perovskite composition. We note that the carrier densities may differ with different quenching efficiencies at the contacts between the samples, and this may in turn influence the subsequent lifetimes.

A major issue hindering the applicability of wide-bandgap perovskites is their phase instability under illumination. To evaluate this, we use a 520 nm continuous-wave laser at 5 sun intensity (300 mW/cm^2^) to excite encapsulated samples and monitor their PL over time. Samples with bandgaps in the range between 1.62 and 1.77 eV show excellent emission stability, with no changes in their PL spectra over time at these photon doses (Figure 2c and Figure S9). In contrast, severe phase segregation occurs in the 1.80 eV perovskite during the first minutes under illumination, potentially linked to the substantial halide heterogeneity observed by nXRF mapping (see Figure 2b). We note that during the evaporation, the substrate holder is rotating to improve the film uniformity, which might benefit the precursor (halide) mixing.

We fabricate single-junction solar cells based on the different perovskite compositions to evaluate their performance when integrated into working devices. We use the device architecture introduced in Figure 1a with MeO-2PACz as the HTM and show the *J*–*V* curves in Figure 3a and EQE spectra in Figure 3b. These measurements demonstrate efficient photocarrier-to-electron conversion for all devices and a blue-shifted absorption onset upon Br addition to the perovskite composition. The device *V*_OC_ monotonically increases for higher perovskite bandgaps (Figure 3c), with the highest *V*_OC_ of 1.24 V being observed for the 1.77 eV evaporated perovskite (Table 1). There is no further voltage gain for a device based on a 1.80 eV bandgap absorber. We associate this *V*_OC_ saturation to the substantial phase segregation (cf. Figure 2b,e), which produces low-gap clusters onto which charge carriers funnel. To gain a further understanding of our device losses, we calculate the estimated *V*_OC,rad_ based on the Urbach fit of EQE spectra to obtain the extended EQE tail for dark current calculation (Figure S10; see Methods for more details) and represent the *V*_OC_ loss associated with the different evaporated compositions in Figure 3d.^43,44^ We observe that the *V*_OC_ loss increases from 196 to 251 mV when we tune the perovskite bandgap from 1.62 to 1.80 eV. The Urbach energy also rises with the bandgap energy from 13.5 to 19.0 meV. These observations indicate higher electronic disorder upon Br addition and are consistent with the increased trap densities revealed from the PL measurements (cf. Figure 2d).^2^ These collective performance and photostability results suggest that the evaporated FA_0.7_Cs_0.3_Pb(I_0.64_Br_0.36_)_3_ perovskite with a 1.77 eV bandgap is our best candidate for use as a front absorber in a tandem architecture with sufficient photostability and high, albeit still suboptimal, *V*_OC_.

**Figure 3:**
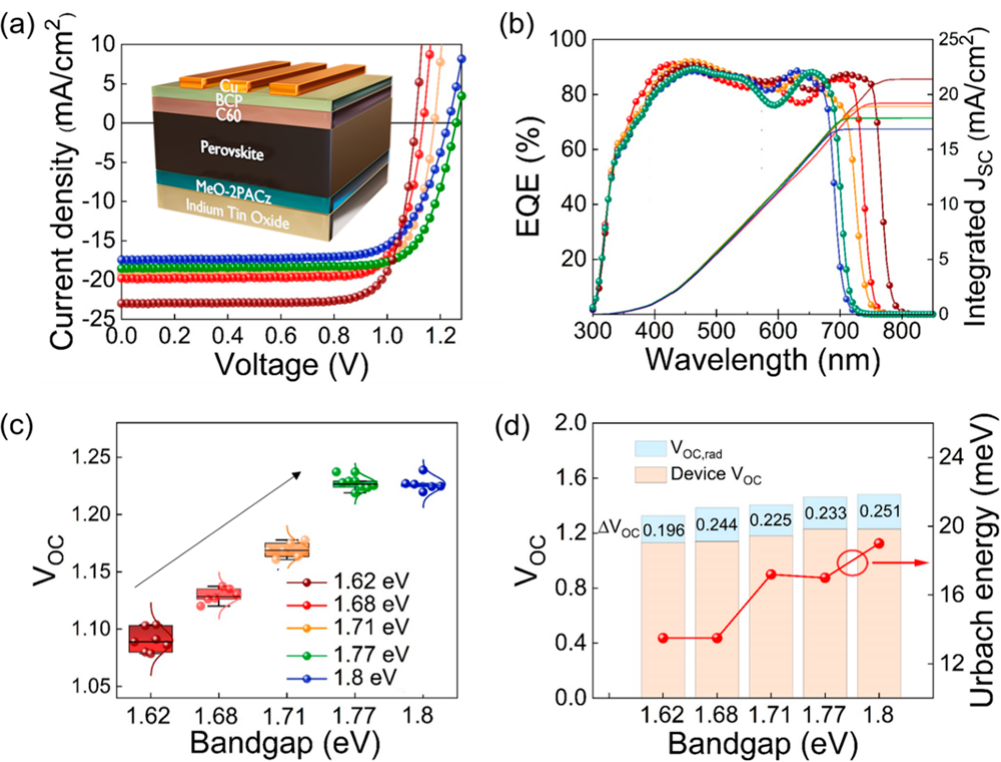
(a) *J*–*V* curves at AM1.5G illumination of evaporated solar cells based on perovskites with different bandgaps with color legend as in (c): 1.62 eV (dark red curve), 1.68 eV (red), 1.71 eV (orange), 1.77 eV (green) and 1.80 eV (blue). See Table 1 for compositions. EQE spectra and the corresponding integrated *J*_SC_ (b) and the *V*_OC_ statistics (c) for the series of devices shown in (a). Box/whisker plots contain the 1.5 interquartile range, the median value, and data distribution of the *V*_OC_. (d) Measured *V*_OC_, calculated *V*_OC_ loss, and Urbach energy extracted from EQE for champion devices based on different bandgap evaporated perovskites.

With the losses associated with the front perovskite interface being minimized by employing an HTM based on MeO-2PACz, we now focus on overcoming the losses arising from the rear perovskite interface. Hu et al. have employed EDAI_2_ as a surface treatment to passivate MA-based Pb/Sn perovskite and achieved an excellent device performance of 23.6%.^45^ Here, we demonstrate that the same approach works well for vacuum-deposited perovskites, in particular to passivate the 1.77 eV evaporated FA_0.7_Cs_0.3_Pb(I_0.64_Br_0.36_)_3_ composition (see Methods). We observe an order-of-magnitude improvement in PLQE from 0.01% to 0.1% after surface passivation of the thin film with EDAI_2_ (Figure 4a), which corresponds to a large reduction of nonradiative losses. We note that PLQE measurements are taken on samples deposited on MeO-2PACz/glass to ensure the perovskite formation is relevant to devices and that SEM images do not show obvious surface roughening which could otherwise promote better light outcoupling (Figure S11). We then thermally evaporate C_60_ on top of the perovskite to have a complete device stack and observe that the PLQE drops to 0.02%. On the contrary, the PLQE of the device stack without EDAI_2_ treatment is below our detection limit (≪0.005%), validating the passivation effect of EDAI_2_ (Figure S12). This result is consistent with previous reports which reveal that interfacial losses between perovskite and C_60_ are severe if unmitigated.^46^ XRD measurements show some incorporation of iodide into the perovskite upon EDAI_2_ passivation (Figure S13),^47^ but no low-angle peak is observed which excludes significant formation of 2D perovskite on the surface as reported by others.^48^ Finally, TRPL measurements show prolonged charge carrier lifetimes in the EDAI_2_-passivated sample (Figure S14), strengthening the viability of the approach to increase charge carrier diffusion lengths in eventual devices under operation.

**Figure 4:**
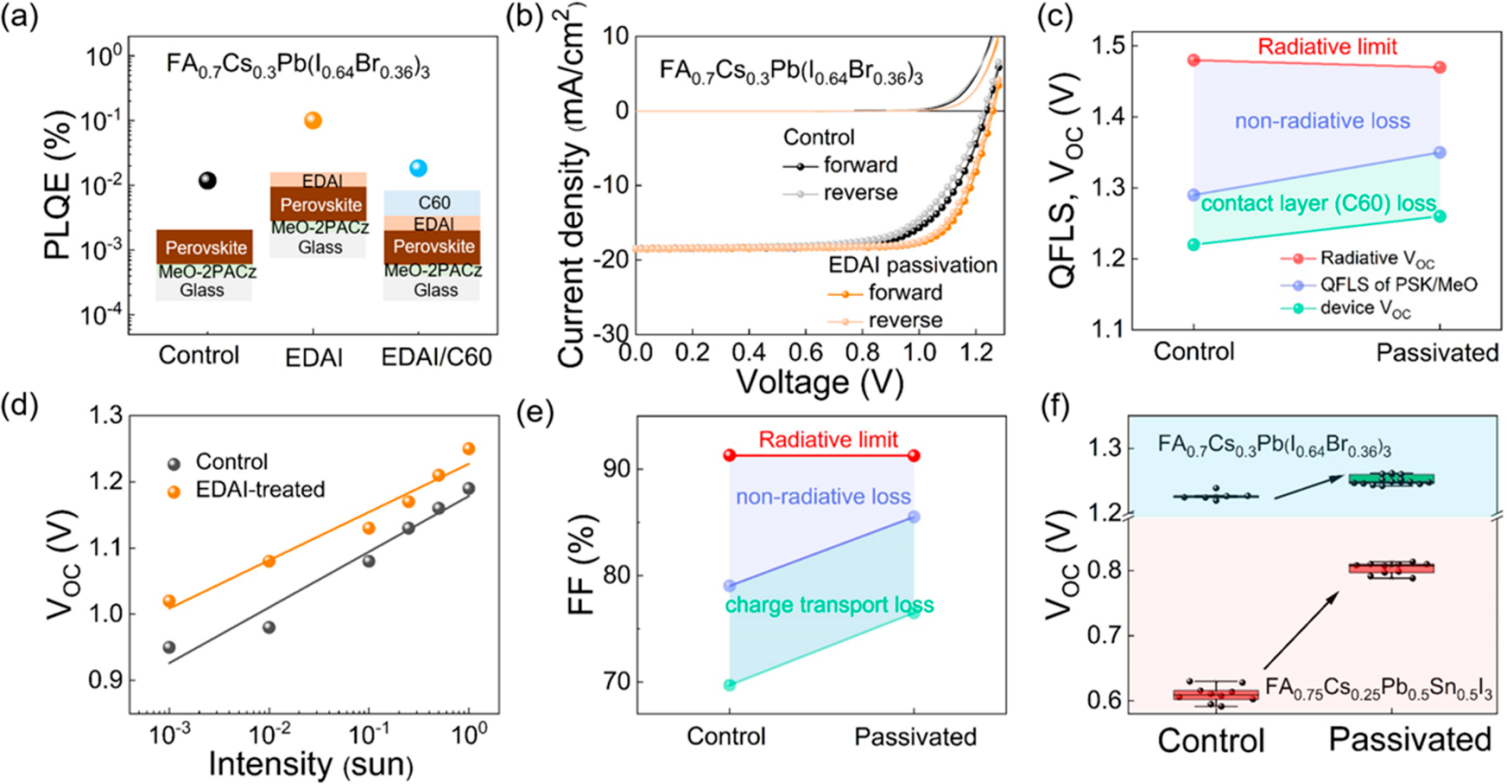
(a) PLQE values for a FA_0.7_Cs_0.3_Pb(I_0.64_Br_0.36_)_3_ (1.77 eV) evaporated perovskite film, with EDAI_2_ treatment and with EDAI_2_ treatment plus C_60_ contact layer. *J*–*V* parameters are shown in Table 2. (b) *J*–*V* curves for control and EDAI_2_-passivated FA_0.7_Cs_0.3_Pb(I_0.64_Br_0.36_)_3_-based solar cells in the dark (no symbols) and under 1 sun AM 1.5 G illumination (line and symbols). See device parameters in Table 2. Radiative limit, pseudo nonradiative loss, and experimental values of the *V*_OC_ (QFLS) (c) and FF (e) for control and EDAI_2_-treated devices. (d) *V*_OC_ as a function of incident light intensity, showing ideality factors of 1.46 and 1.27. (f) *V*_OC_ statistics for devices based on FA_0.7_Cs_0.3_Pb(I_0.64_Br_0.36_)_3_ and FA_0.75_Cs_0.25_Pb_0.5_Sn_0.5_I_3_ with and without EDAI_2_ passivation.

We fabricate solar cells where the 1.77 eV evaporated perovskite is passivated with EDAI_2_ and show the *J*–*V* curves in Figure 4b. We observe a substantial improvement in *V*_OC_ and fill factor (FF) with respect to the unpassivated sample. In particular, the *V*_OC_ reaches a very high value of 1.26 V (Figure S15). Figure 4c shows a comparison between the quasi-Fermi level splitting value extracted from PLQE data (see Methods for details on the calculations) and the actual device *V*_OC_ for both the control and passivated samples. A difference between those values relates to the relative importance of intrinsic perovskite nonradiative recombination processes and the effect of the additional interface introduced by the C_60_ layer with energetic offsets.^49^ Interestingly, EDAI_2_-treated devices gain 40 mV with respect to the control device as a result of surface passivation, following the trend observed in PL. Further analysis of the EQE curves shows a concomitant reduction in Urbach energy from 17.0 to 15.5 meV (Figure S16). We note that the perovskite bandgap slightly reduces from 1.77 to 1.76 eV as a result of iodine incorporation upon EDAI_2_ passivation (Figure S17), consistent with our XRD results.

To better understand the passivation effect on the FF, we conduct a light-intensity-dependent measurement of the *V*_OC_ and extract the ideality factor (Figure 4d). Using these data to extract pseudo-*J*–*V* curves (see Methods and Figure S18),^50^ we deconvolute the effect of charge transport and nonradiative losses within the devices, showing 79.0% and 85.5% FF when there is no charge transport loss. Figure 4e summarizes the results, highlighting the reduction in the nonradiative losses for the EDAI_2_-passivated samples. In contrast, we have identified an absolute reduction in FF by 10% from charge transport losses in both control and EDAI_2_-passivated samples, indicating that further optimization is still required to find ideal contact layers. The EDAI_2_ passivation strategy also better stabilizes the device *V*_OC_ under light soaking compared to the unpassivated control (Figure S19). We observe a 1.4% absolute increase in average PCE for the EDAI_2_-passivated solar cells compared to the control when comparing batch-to-batch variation (Figure S20), demonstrating the reproducibility of EDAI_2_ passivation.

In order to demonstrate a narrow-bandgap subcell suitable for a tandem configuration, we first develop devices based on an ITO/2-PACz/FA_0.75_Cs_0.25_Pb_0.5_Sn_0.5_I_3_/C60/BCP/Cu architecture, where the perovskite in this case is deposited by solution processing. For the perovskite, we observe a grain size of around 500 nm, PL emission at 970 nm, and bandgap at 1.28 eV (Figure S21). EDAI_2_ passivation substantially improves the *V*_OC_ in these narrow-bandgap perovskite solar cells as previously reported,^45^ increasing the champion PCE from 12.6% to 19.4% (Figure 4f, Figure S22, and Table 2) and average from 11.2% to 18.4% (Figure S23).^45^ We note that we do not see any morphology variation or EQE onset shift after EDAI_2_ passivation (Figures S24 and S25, respectively). We see a reduction in *V*_OC_ loss from 382 to 140 mV and Urbach energy from 21.5 to 20 meV (Figure S26) of the perovskite absorber when comparing devices without and with EDAI_2_ passivation, respectively, consistent with reduced nonradiative loss and electronic disorder in the perovskite films after EDAI_2_ treatment. We note that vapor-deposited EDAI_2_ has also been reported to result in enhanced performance, though passivation is more robust using the solution approach herein presented for the EDAI_2_ treatment.^51^

**Table 2:** Champion PV Performance Metrics for Evaporated Wide-Bandgap (1.77 eV) and Solution-Processed Narrow-Bandgap (1.28 eV) Perovskite Solar Cells with and Without EDAI_2_ Treatment and 2-Terminal All-Perovskite Tandem Solar Cells^a^

	*V*_OC_ (V)	*J*_SC_ (mA/cm^2^)	FF (%)	PCE (%)	*V*_OC_ loss (mV)
Wide-Bandgap (1.77 eV/1.76 eV) Perovskite Solar Cells					
control	1.24	–18.5	69.7	16.0	230
EDAI_2_-treated	1.26	–18.5	76.5	17.8	190
Narrow-Bandgap (1.28 eV) Perovskite Solar Cells					
control	0.64	–30.6	64.3	12.6	380
EDAI_2_-treated	0.86	–32.0	70.6	19.4	140
All-Perovskite Tandem Solar Cell					
	2.06	–15.2	76.9	24.1	

a The *V*_OC_ loss calculation is based on the equation of *V*_OC,rad_ – *V*_OC_, where ; see details in Methods.

We build two-terminal monolithic all-perovskite tandem solar cells based on our optimized evaporated wide gap (1.77 eV) and solution-processed narrow-bandgap (1.28 eV) perovskite subcells, with a SnO_*x*_ interconnection layer deposited by atomic layer deposition (ALD).^52,53^ We note that the ALD-SnO_*x*_ does not affect the performance of the wide-bandgap subcell (Figure S27). The architecture of the tandem device is shown in Figure 5a and is comprised of ITO/MeO-2PACz/FA_0.7_Cs_0.3_Pb(I_0.64_Br_0.36_)_3_/EDAI_2_/C60/ALD-SnO_*x*_/Au/PEDOT:PSS/FA_0.75_Cs_0.25_Pb_0.5_Sn_0.5_I_3_/EDAI_2_/C60/BCP/Cu. An ∼1 nm Au cluster layer is used between the SnO_*x*_ and the PEDOT:PSS layers to improve charge recombination and enhance device *V*_OC_ and FF.^52^ We also note that the choice of Au (as opposed to Cu, for instance) for the recombination junction is important to ensure good charge transport (Figure S28 and Table S2). We found that employing 2PACz for the narrow-bandgap subcell in a tandem architecture has a negative impact on charge transport, which we attribute to phosphoric acid groups not anchoring well to the Au clusters.^54^ Therefore, we utilize PEDOT:PSS instead of 2PACz. Figure 5b displays an SEM cross section image of the tandem device under study, where the thicknesses of the wide-bandgap and narrow-bandgap perovskites are 300 and 800 nm, respectively, to match the current of each subcell. Figure 5c shows the *J*–*V* curves of the champion all-perovskite tandem solar cell, showing a PCE of 24.1%, with an excellent *V*_OC_ of 2.06 V, a *J*_SC_ of 15.2 mA/cm^2^, and a FF of 76.9% from the forward scan direction and negligible hysteresis between the forward and backward scans. The integrated J_SC_ values extracted for the wide-bandgap and narrow-bandgap subcells from the EQE spectra are 14.5 and 14.9 mA/cm^2^, respectively (Figure 5d). This PCE is the highest reported so far for all-perovskite tandem solar cells where at least one perovskite absorber is prepared by vacuum deposition. Comparing to the sum *V*_OC_ of the champion subcells, the tandem device shows a small voltage loss of 60 mV, which we attribute to the interconnecting layer that requires further optimization. We observe a stabilized performance output PCE of 23.2% at a fixed bias of 1.74 V (Figure S29). The device statistic data for the PCE and the *V*_OC_, and *J*_SC_, and FF across four batches are displayed in Figure 5e,f, showing a standard deviation of 1.5% in PCE, thus confirming reasonable batch-to-batch reproducibility. Our encapsulated devices kept in air show excellent shelf life stability, retaining 92% of their initial PCE after 26 days (Figure S30).

**Figure 5:**
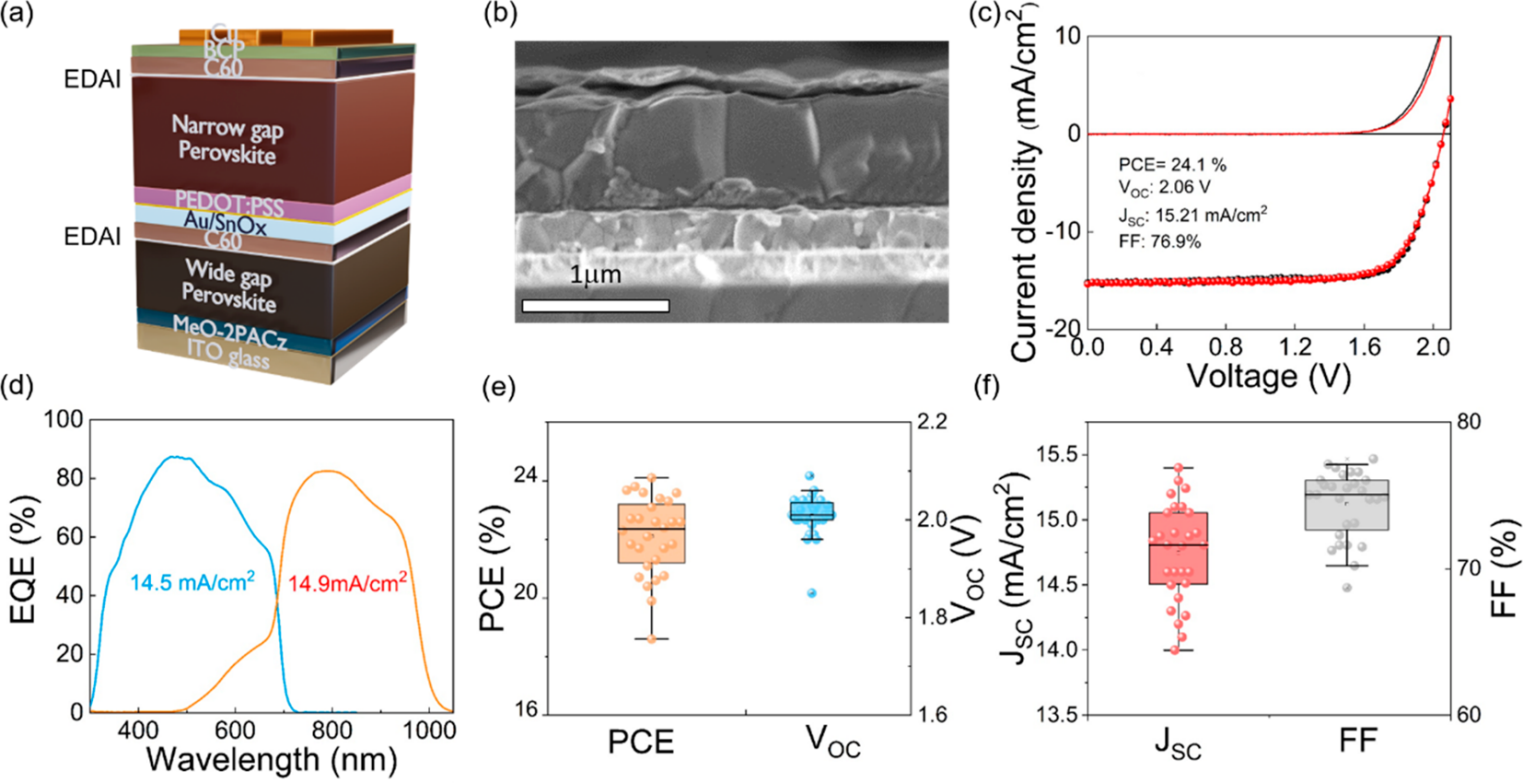
(a) Schematic and (b) cross-sectional SEM image displaying the architecture of the all-perovskite tandem solar cell. (c) *J*–*V* curves of the champion tandem device, reaching a 24.1% PCE for forward scan. (d) EQE spectra of the evaporated-based wide-bandgap (blue curve) and solution-based narrow-bandgap (orange) perovskite subcells comprising the all-perovskite tandem device. (e, f) Distribution of device performance across four batches (28 devices) with an average PCE of 22.1%.

In summary, our work employs a 4-source vacuum deposition method to demonstrate FA_0.7_Cs_0.3_Pb(I_*x*_Br_1–*x*_)_3_ perovskites with a tunable bandgap. Engineering the device architecture via use of a MeO-2PACz layer as the HTM demonstrates a 20.7% PCE in a 1.62 eV bandgap perovskite solar cell, which is the highest value for a MA-free device performance in a multisource evaporated system to date. Several evaporation sources enable fine-tuning of the halide content, and we use it to report a phase-stable FA_0.7_Cs_0.3_Pb(I_0.64_Br_0.36_)_3_ with a 1.77 eV bandgap and minimized nonradiative losses when treated with EDAI_2_. This passivation method is versatile and reproducible, and we extend it to Pb/Sn-based narrow-bandgap perovskite solar cells to build a 2-terminal tandem solar cell that shows a PCE of 24.1% with an excellent *V*_OC_ of up to 2.06 V. Our result is a first step toward all-vapor-deposited tandems and encourages future work to develop narrow-bandgap perovskites benefiting from the scalable, conformal, and reproducible character of vacuum deposition methods. These systems open a myriad of possibilities for enhanced modularity including exploration of new recombination layers not compatible with solution-processed perovskites and integration of advanced photonic strategies to push perovskite photovoltaics to their limits.

## Data Availability

The data and code that support the findings of this study are available at https://doi.org/10.17863/CAM.96734 in the University of Cambridge Apollo repository.

## Supporting Information Available

Materials and methods for evaporated perovskite solar cells, solution-processed low-gap perovskite solar cells, and all-perovskite tandem devices, methods for characterization of solar cell performance, SEM, XRD, PLQE, nXRF, EQE, and TCSPC, radiative *V*_OC_ and Urbach energy calculations, SEM, PL, XRD, and device performance for solar cells based on different HTMs, solar cells based on perovskites with different bandgaps, and effect of EDAI_2_ passivation, and interlayer optimization and stability data for all-perovskite tandem solar cells (PDF)
