# Supplementary material for: Vacuum-Deposited Wide-Bandgap Perovskite for All-Perovskite Tandem Solar Cells
Source: ACS Energy Lett. 2023 May 24;8(6):2728–37. doi: 10.1021/acsenergylett.3c00564 (PMC10262197; doi:10.1021/acsenergylett.3c00564)
Supplement: Supplementary file 1 — nz3c00564_si_001.pdf [file nz3c00564_si_001.pdf]

Supporting information

Vacuum-deposited wide-bandgap perovskite for all-perovskite tandem solar cells

Yu-Hsien Chiang,[a] Kyle Frohna,[a] Hayden Salway,[b] Anna Abfalterer,[a] Linfeng Pan,[a] Bart Roose,[b] Miguel Anaya,[*b] Samuel D. Stranks[*a.b]

a. Cavendish Laboratory, Department of Physics, University of Cambridge, JJ Thomson Avenue, Cambridge CB3 0HE, United Kingdom

b. Department of Chemical Engineering & Biotechnology, University of Cambridge, Philippa Fawcett Drive, Cambridge CB3 0AS, United Kingdom

Methods

Materials

Formamidinium iodide was purchased from Greatcell solar. Caesium bromide (99.999% trace metals basis, 203017), Tin iodide (beads, 99.99%, 409308), Tin flouride (99%, 334626), Caesium iodide (99.999% trace metals basis, 203033), N,N-Dimethylformamide (anhydrous, 99.8%, 227056), Dimethyl sulfoxide (anhydrous> 99.9%, 276855), PTAA, Toluene (anhydrous, 99.8%, 244511), Isopropanol (anhydrous, 99.5%, 278475) and Ethane-1,2-diammonium iodide (>99.8%, 900852) were purchased from Sigma-Aldrich. Lead iodide, Lead bromide (for perovskite sublimation), 2PACz and MeO-2PACz were purchased from TCI. Lead iodide for solution lead-tin samples was purchased from Alfa Aesar (ultra-dry 99.999% metals basis). Poly(3,4-ethylenedioxythiophene):polystyrene sulfonate (PEDOT:PSS) was purchased from Heraeus, LLC (CLEVIOS™ P VP AI 4083). Gold was purchased from KJ Lesker. BCP and C60 were purchased from Ossilla and Creaphys. Tetrakis(dimethylamino)Sn (TDMASn) was purchased from Epivalence.

Device fabrication

Evaporated perovskite solar cells

ITO glass (1 inch x 1 inch, 15 ohm/cm^2, Kintec) was cleaned in the sonication bath with the following steps for 15 minutes each, DI water, acetone and isopropanol. The cleaned substrate was transferred to UV-ozone chamber (UVC1014, NanoBioAnalytics) for another 15 minutes post-treatment. 0.6 M MeO-2PACz in ethanol was dropped on the cleaned ITO and spun at 3000 r.p.m. for 30 seconds (with the lid open) in an M-Braun glovebox with integrated spin-coater, following by post-annealing at 100 ^{o}C for 10 minutes. The MeO-2PACz substrates were transferred to a PEROevap (CreaPhys) chamber inside a N_2-filled glovebox for perovskite evaporation. After the perovskite evaporation, post-annealing is required at 135 ^{o}C for 90 minutes (1.62 eV sample) or 30 minutes (other evaporated perovskites). For

surface passivation, $EDAI_2$ (0.5 mg/ml) was dissolved in mixed IPA and toluene (volume ratio 1:1) at 70 °C heating and stirring for 2 hours. The solution was filtered with 0.22 µm pore size. 120 µl of $EDAI_2$ solution was quickly dropped onto the perovskite film and spun at 4000 r.p.m. for 20 seconds. The film was annealed at 100 °C for 5 minutes. To complete the single-junction device, the samples were back-transferred to the perovskite evaporator for C60 (25 nm), BCP (8 nm) and Cu (120 nm) deposition.

Perovskite evaporation

The cleaned ITO substrates were transferred to the perovskite evaporator in the glovebox. During the evaporation, the substrates stage was kept at around 18 °C temperature, while the chamber wall was at -15 to -20 °C temperature. To achieve the bandgap of 1.62 eV, the precursor deposition rates were 1 Å/s for FAI, 0.6 Å/s for PbI_2 and 0.1 Å/s for CsBr. For the widegap perovskite, the fourth source $PbBr_2$ was employed for multisource evaporation. For the 1.77 eV bandgap perovskite, the deposition rates were 1 Å/s for FAI, 0.6 Å/s for PbI_2, 0.2 Å/s for $PbBr_2$ and 0.1 Å/s for CsBr. We set the tooling factor of PbI_2 and $PbBr_2$ to 93.6 % and 115.2 % to fine tune the deposition rate and get the rate of 0.434 Å/s for PbI_2, 0.156 Å/s. for $PbBr_2$. The FAI, PbI_2 and $PbBr_2$ powder was refilled every time before evaporation. The distance between sources to substrate holder is around 35 cm. During deposition, the working pressure was between 1 to $4x10^{-6}$ mbar.

Solution-processed low band gap perovskite solar cells

The cleaned ITO substrates after UV-ozone were transferred into the glovebox for 2PACz deposition. 10 mM 2PACz solution in ethanol was dropped onto the substrate and spun at 2000 r.p.m. for 30 seconds (with the lid open), with a post-annealing at 100 °C for 10 mins. The substrates were twice washed with ethanol at 5000 r.p.m. for 30 seconds (dynamic dropping of 120 µl ethanol during this process) and the substrate heated at 100 °C for 5 mins. To make the perovskite solution, the precursors were mixed in one vial in the following order, SnF_2 (0.1 M), SnI_2 (1 M), CsI (0.5 M), PbI_2 (1 M) and FAI (1.5 M) to

achieve 2 M concentration in DMF:DMSO (4:1) for the composition of $FA_{0.75}Cs_{0.25}Pb_{0.5}Sn_{0.5}I_3$. The low-gap perovskite solution was prepared in a N_2-filled glovebox (H_2O and O_2 below 1 ppm) and stirred for 3 hours before use. 120 µl perovskite solution was spread on ITO and spun at 5000 r.p.m. for 40 s. The samples were moved to a hotplate for post-annealing at 120 ºC for 10 mins. The $EDAI_2$ surface passivation method is the same as above.

All-perovskite tandem solar cells

The widegap perovskite was deposited by vacuum deposition. The procedure is the same as above until C60 layer deposition. After that ALD-SnO_x deposition was employed with (TDMA)Sn and deionised water H_2O as precursors. The base chamber pressure is around 10 mbar and the deposition recipe of t1/t2/t3/t4 of TDMASn pulse/ N_2 purge/ H_2O pulse/ N_2 purge time are 0.6 s, 10 s, 0.1 s and 10 s for 200 cycles (target thickness: 20 nm SnO_x). The temperature of source bottle, line and substrate were kept at 75, 90 and 100 ºC. The boost system is on with N_2 as carrier gas to increase the vapour of TDMASn. After the deposition, the samples were transferred to the evaporator for 1 nm Au deposition. The diluted PEDOT:PSS (1:3 v:v in methanol) was spun on the substrates with 4000 r.p.m. for 30 seconds with a ramp time of 1000 r.p.m./s and the deposited films were annealed at 120 ºC for 20 mins in air. After annealing, the samples were transferred to the glovebox immediately for PbSn perovskite deposition and the whole device fabrication.

Solar cells characterisation

The wide bandgap perovskite solar cells were measured under 1 sun AM 1.5G condition with a calibrated KG5 filter reference cell by a Xenon lamp from Abet Sun 2000 Solar Simulators (AAB class). For narrow bandgap and tandem perovskite solar cells and all-perovskite tandem solar cell, a KG2 filter reference cell was used to calibrate the light intensity. We used Sunbrick G2V LEDs solar simulator with AAA class for all-perovskite tandem solar cells measurement. The spectral mismatch of solar

simulator is <5%. The solar cells measurement were conducted in air with no temperature control system. Most of the devices are encapsulated (UV-curable epoxy) in N_2-filled glovebox before test. We noticed that the device V_{OC} is slightly higher with encapsulation. During the JV scan, both forward and reverse scan were recorded with a scan speed of 100 mV/s. The solar cells active area is 13.8 mm^2, defined by the overlap area between ITO and Cu contact. A measurement mask with 8.1 mm^2 size was used during the measurement.

Intensity dependent Sun-V_{OC} measurement was measured by different neutral density filters to attenuate the light intensity for JV-scans. The Pseudo_JV curve was obtained by

$$PJ(I) = J_{SC,1sun} - J_{SC}(I) \cdots\cdots\cdots\cdots\cdots \quad \cdots\cdots\cdots\cdots\cdots\cdots (1)$$

Since the measurement was conducted under open-circuit condition, the net charge is zero in the cell, which can calculate the pseudo FF when there is no charge transport loss.

Radiative V_{OC} and Urbach energy calculation[1]

To understand the maximum power conversion efficiency of a semiconductor with a given bandgap, the Shockley-Queisser model is used to define radiative recombination by the detailed-balance principle.

$$V_{\mathrm{OC}} = \frac{k_{\mathrm{B}}T}{q}\ln\left(\frac{J_{\mathrm{SC}}}{J_0}\right) \cdots\cdots\cdots\cdots\cdots\cdots\cdots \quad \cdots\cdots\cdots\cdots\cdots\cdots (2)$$

where q is element charge, k_{B} is Boltzmann constant, T is temperature, J_{SC} is short-circuit current, J_0 is dark saturation current. The expressions of J_{SC} and J_0 are given by:

$$J_{\mathrm{SC}} = q\int_0^{\infty} \mathrm{EQE}_{\mathrm{PV}}(E)\phi_{\mathrm{AM1.5}}(E)\, dE \cdots\cdots\cdots\cdots\cdots\cdots\cdots\cdots\cdots\cdots\cdots\cdots\cdots (3)$$

$$J_0 = \frac{q}{EQE_{\mathrm{EL}}}\int_0^{\infty} \mathrm{EQE}_{\mathrm{PV}}(E)\phi_{\mathrm{BB}}(E)\, dE \cdots\cdots\cdots\cdots\cdots\cdots\cdots\cdots\cdots\cdots\cdots\cdots\cdots (4)$$

Where $\phi_{\mathrm{BB}}(E) = \frac{2\pi E^2}{h^3 c^2} \frac{1}{\exp\left(\frac{E}{k_{\mathrm{B}}T}\right) - 1}$, ϕ_{BB} is the blackbody spectrum at room temperature

In the radiative limit, we set $\mathrm{EQE_{EL}}$ to 1,

$$V_{OC,rad} = \frac{k_B T}{q} \ln\left(\frac{\int \mathrm{EQE_{PV}}(E)\,\phi_{\mathrm{AM1.5}}(E) dE}{\int \mathrm{EQE_{PV}}(E)\phi_{\mathrm{BB}}(E)\, dE}\right) \cdots\cdots\cdots\cdots\cdots\cdots \text{(5)}$$

SEM

The surface morphology of the perovskite thin films was observed using a field-emission scanning electron microscope (ZEISS LEOGEMINI 1530VP FEG-SEM) with a beam energy of 2 kV, an in-lens detector and secondary electron detection mode. The samples were mounted by carbon tape on the holder.

XRD

A Bruker D8 ADVANCE system with a Copper focus X-ray tube (Ka: 1.54 Å) was used to obtain the XRD patterns with operation voltage of 40 kV. During the measurement, the samples were kept in air. The scan range for 2θ was from 5° to 55° with a step size of 0.01° and a dwell time of 0.15 s per step. The measurement was conducted in air for widegap perovskite and in an air-tight sample holder (sealed in glovebox) for PbSn perovskite films. We calculate the stoichiometry of the evaporated perovskite film based on our previous work.[2] In general, we prepared solution-processed films with different amounts of A and X components. With Vegard's law for composition ranges in the same phase, the evolution of perovskite peak shift is based on the composition variation, therefore we can acquire a composition map versus XRD peak position

PLQE

PLQE measurements were conducted on encapsulated samples using 520 nm-continuous wave laser excitation under a 60 W/cm^2 (1 sun equivalent intensity). The procedures for measuring and calculating PLQE were as described in a previous work.[5] The samples were mounted in an integrating sphere to

collect the PL emission with an optical fibre coupled to an Andor iDus Si detector. All the samples were encapsulated with UV-curable expoy inside the glovebox before the measurement. The Quasi Fermi Level Splitting (QFLS) calculation is based on the following.

$$QFLS = k_{\mathrm{B}}T\ln\left(PLQE\,\frac{J_{\mathrm{sc}}}{J_{0,\mathrm{rad}}}\right) \cdots\cdots\cdots\cdots\cdots\cdots (6)$$

$$J_{sc} = q\int EQE_{PV}(E)\emptyset_{AM1.5\,G}\,(E)dE \cdots\cdots\cdots\cdots\cdots\cdots (7)$$

$$J_{0,rad} = q\int \alpha(E)\emptyset_{\,BB}\,(E)dE \cdots\cdots\cdots\cdots\cdots\cdots (8)$$

Where α is the absorption coefficient.

nXRF

Synchrotron nXRF measurements were performed at the I14 hard X-ray nanoprobe beamline at the Diamond Light Source, Didcot, UK, the complete setup is described elsewhere.[40] The procedure is as described in ref.[2] X-rays are monochromated from the output of an undulator source to produce a 14 keV beam. The beam is focused using Kirkpatrick-baez mirrors along the 186 m beampath to produce a beam with full-width at half-maximum of ~50 nm at the focal point. The sample is placed on a raster scanning stage and is scanned across this focal point. The energy resolved nXRF signal is collected by a four-element silicon drift detector in back-scattering geometry. Data was analysed in Python, in particular utilising the open source package Hyperspy.[6]

EQE measurement

A Bentham PVE300 system equipped with dual lamps of xenon-quartz and tungsten halogen lamps was used. A silicon reference cell was used to measure the signal response for calibration. For widegap perovskite (narrowgap) solar cells, the response scan was obtained from a spectral range of 300 to 850 (1100) nm with a step size of 5 nm. For tandem solar cells, a green LED bias with 530 nm emission was used for low bandgap subcell measurement and an infrared LED bias with 940 nm emission was

used for wide bandgap subcell measurement. During all measurements, a transformer mode (Bentham S400 474) with a frequency of 300 Hz was employed for Si calibrated cell and perovskite solar cells.

TCSPC

Time-resolved photoluminescence (TRPL) spectra were obtained using time correlated single photon counting (TCSPC) on a FLS1000-dd-stm fluorescence spectrometer (Edinburgh Instruments). The samples were excited using a 448.4 nm pulsed laser (Edinburgh Instruments, HPL-450) with a repetition rate of 200 kHz and 500 kHz for 1.62 eV bandgap perovskite and 1.77 eV bandgap perovskite and pulse width of 102.1 ps. An emission monochromator bandwidth of 2 nm was used and a 680 nm long pass filter employed to prevent scattered light entering the UV-VIS PMT-980 detector.

In Figure 2d, the quick decay in the first 40 ns is not considered for the fittings as it is attributed to quenching by the contacts. The subsequent PL decay is fitted with mono-exponential functions given the low excitation fluence and as a means to provide comparison between the samples:

y = A*exp(-(x-x0)/t1), where A= 0.122 (0.028), x0=47.4 (56.6) ns and t1=84 (395) ns for the 1.77 eV (1.62 eV) evaporated perovskite deposited on top of the MeO-2PACz/ITO contact.

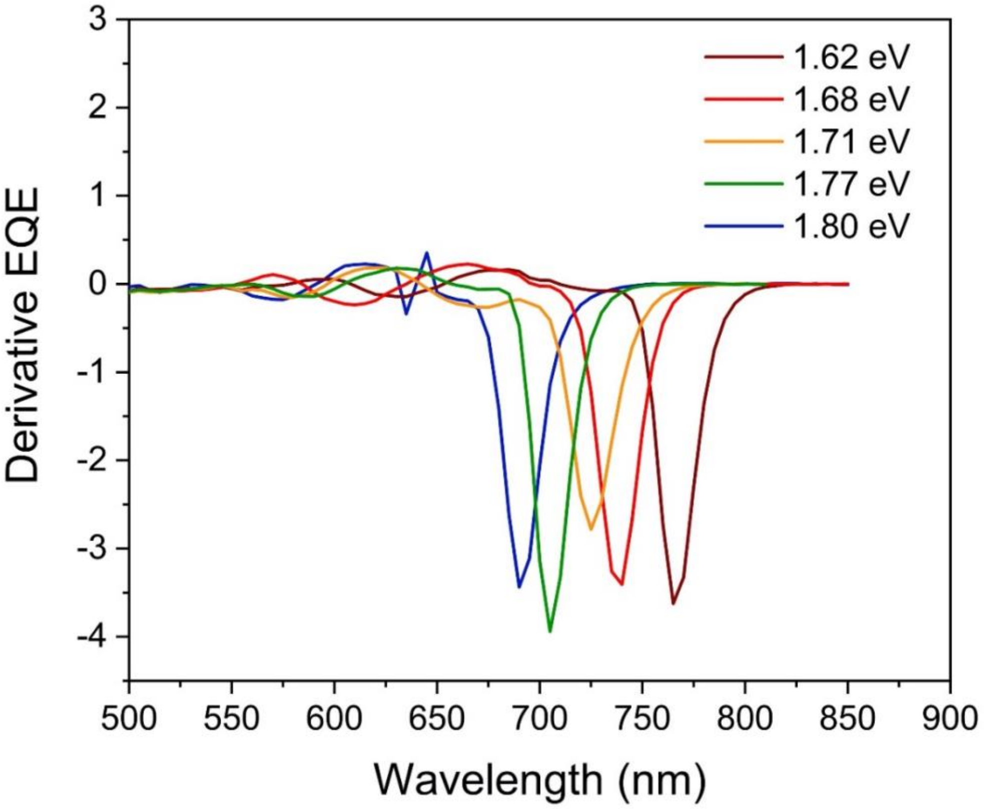

Figure S1. The first derivative of EQE with different bandgap perovskite solar cells.

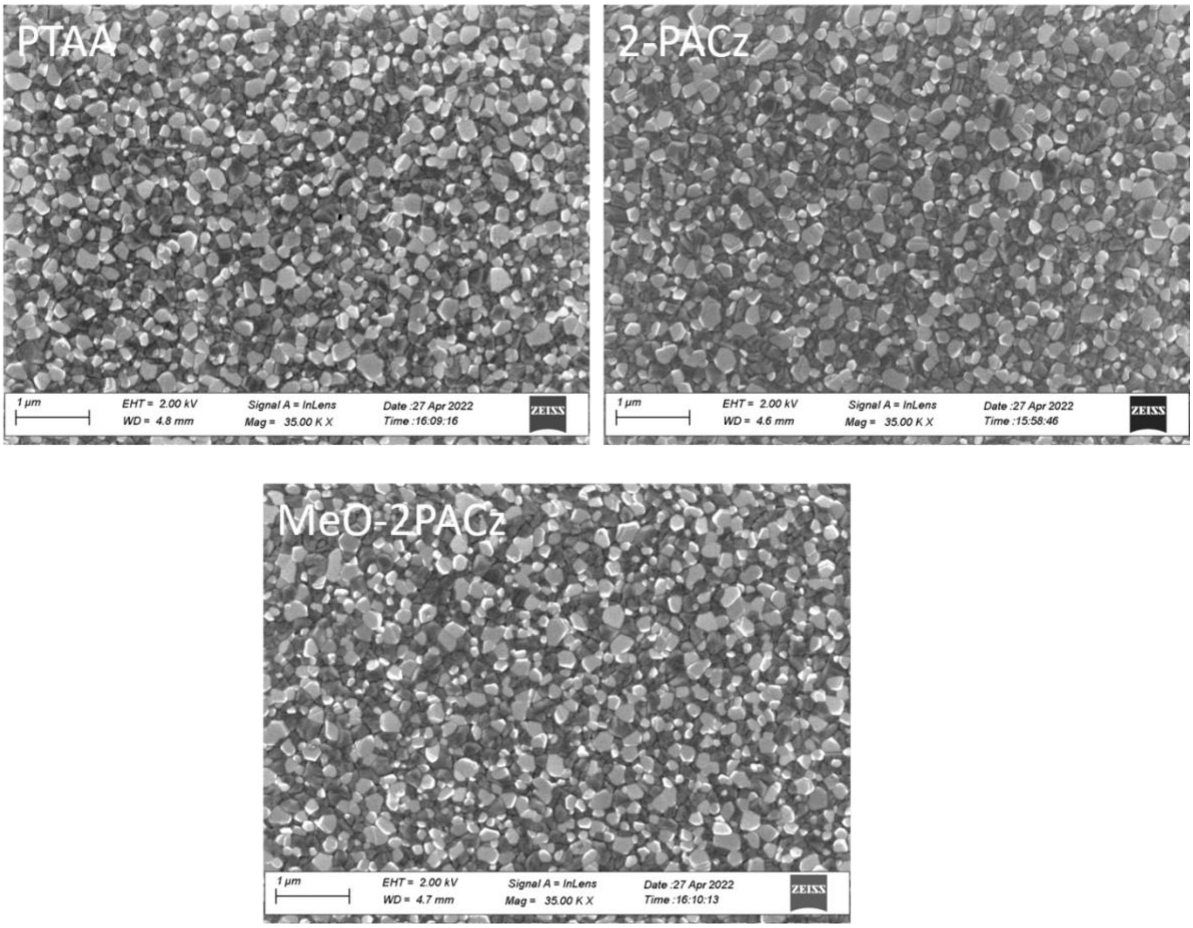

Figure S2. SEM comparison between evaporated perovskite (1.62 eV) film on PTAA, 2PACz or MeO-2PACz layer.

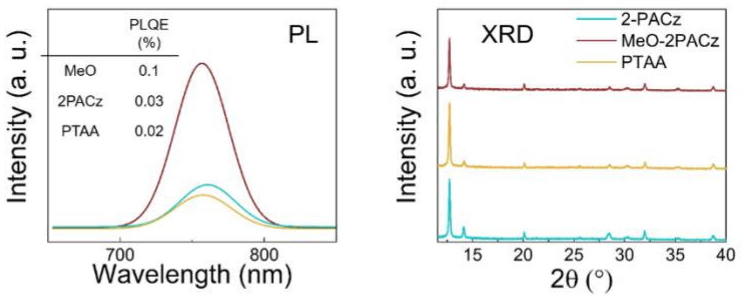

Figure S3. PL(QE) and XRD comparison of evaporated perovskite (1.62 eV) film on top of 2PACz, MeO-2PACz or PTAA layer.

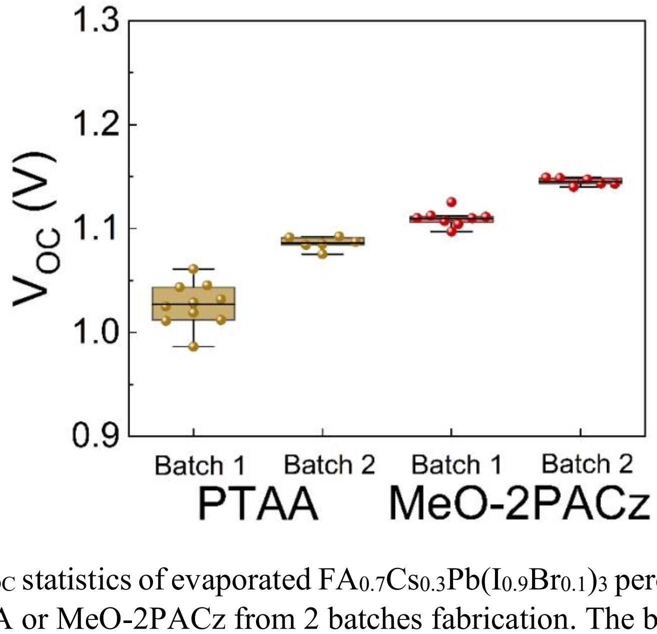

Figure S4. The V_{OC} statistics of evaporated $FA_{0.7}Cs_{0.3}Pb(I_{0.9}Br_{0.1})_3$ perovskite solar cells ($PbBr_2$ rate is 0) on PTAA or MeO-2PACz from 2 batches fabrication. The box/whisker plot contains the 1.5 interquartile range, the median value.

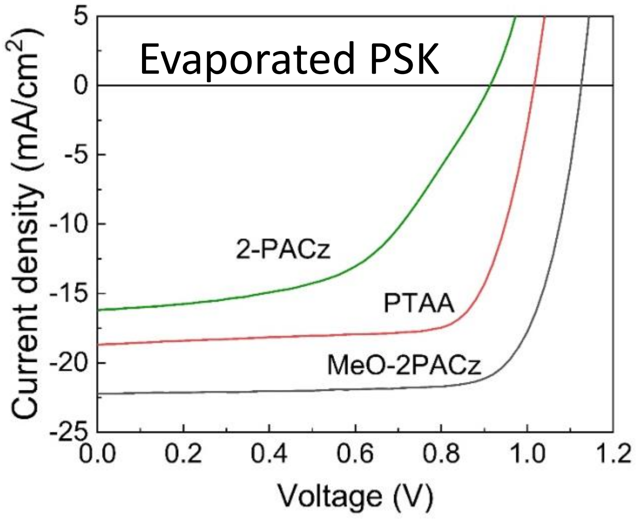

Figure S5. JV curves of evaporated perovskite (1.62 eV, $FA_{0.7}Cs_{0.3}Pb(I_{0.9}Br_{0.1})_3$) on PTAA, MeO-2PACz and 2PACz HTMs.

Table S1, The champion device parameter of evaporated perovskite (1.62 eV, $FA_{0.7}Cs_{0.3}Pb(I_{0.9}Br_{0.1})_3$) on different HTMs.

	V_{OC} (V)	J_{SC} (mA/cm^2)	FF (%)	PCE (%)
PTAA	1.00	-18.69	75.10	14.05
MeO-2PACz	1.10	-22.23	76.63	19.17
2PACz	0.91	-16.20	53.25	7.85

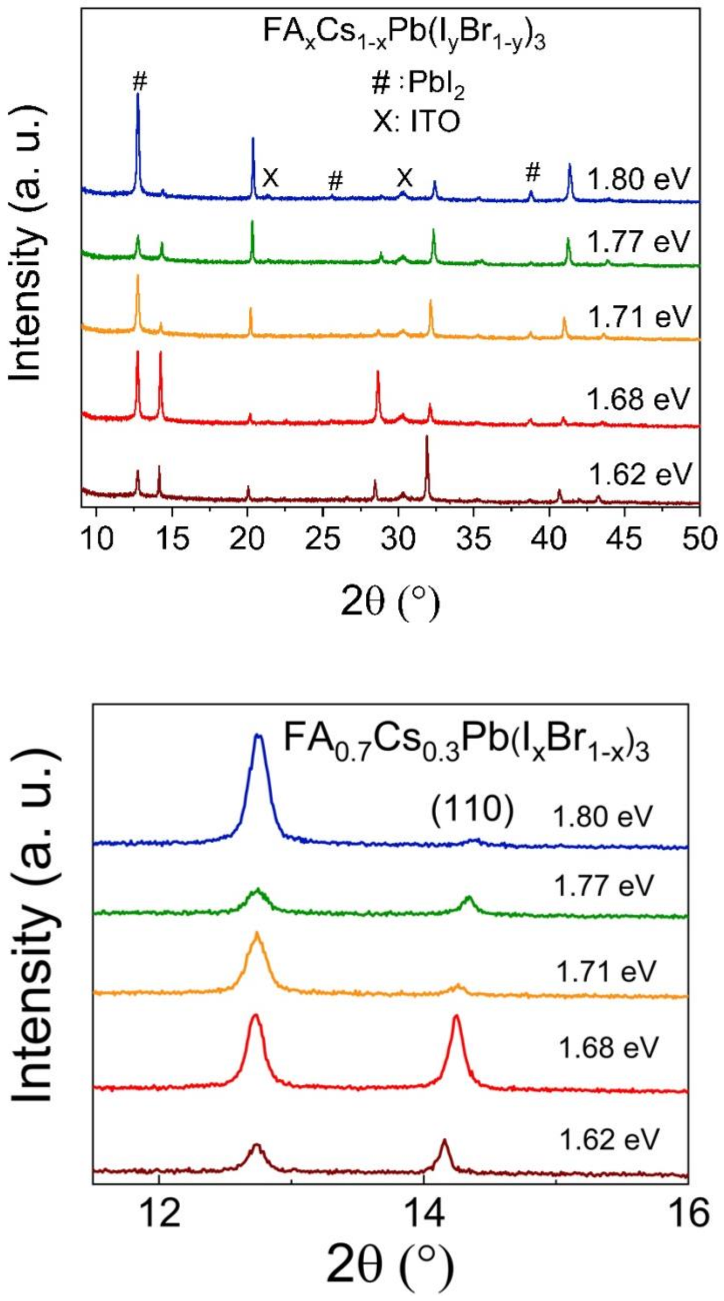

Figure S6. Top: Full region of XRD pattern for different bandgap evaporated perovskite films. Bottom: zoom-in highlighting peak shifting upon addition of Br.

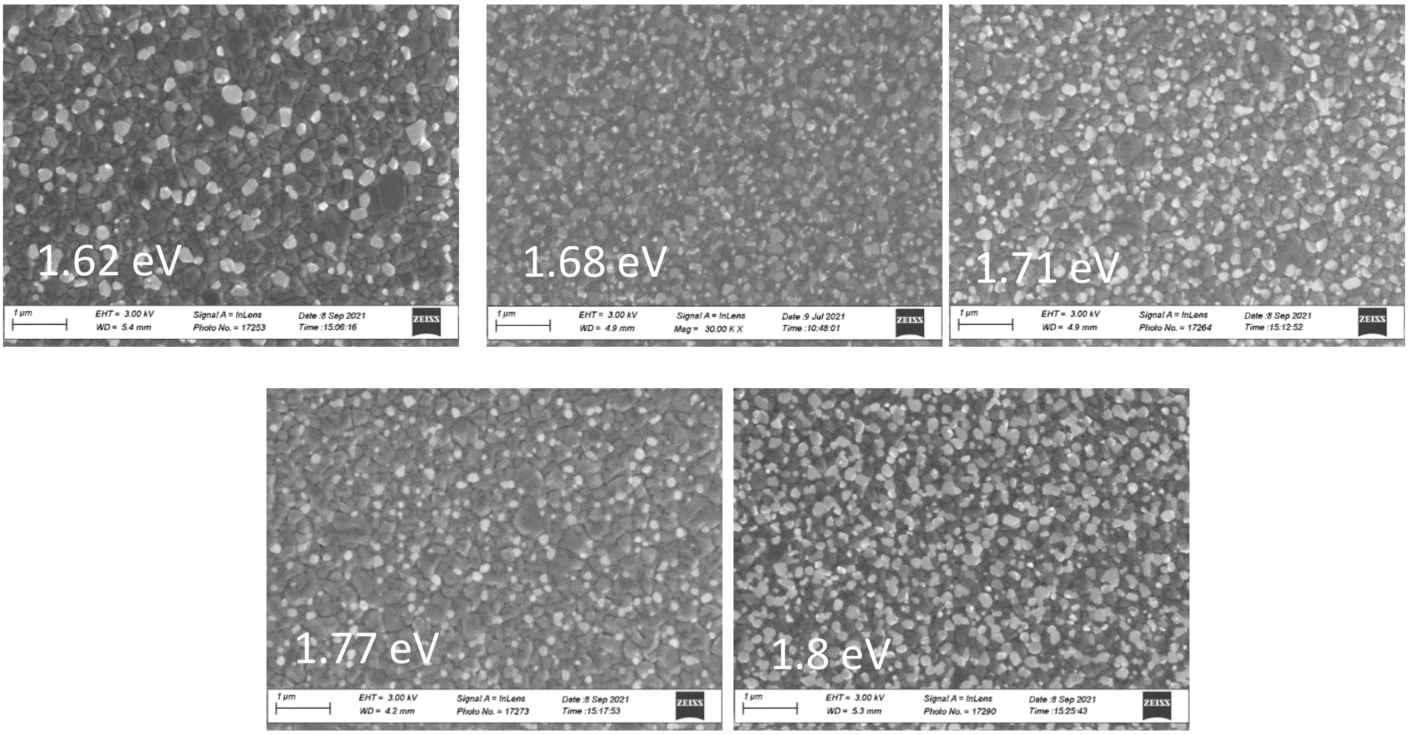

Figure S7. The SEM morphology of different bandgap evaporated perovskite on MeO-2PACz/ITO.

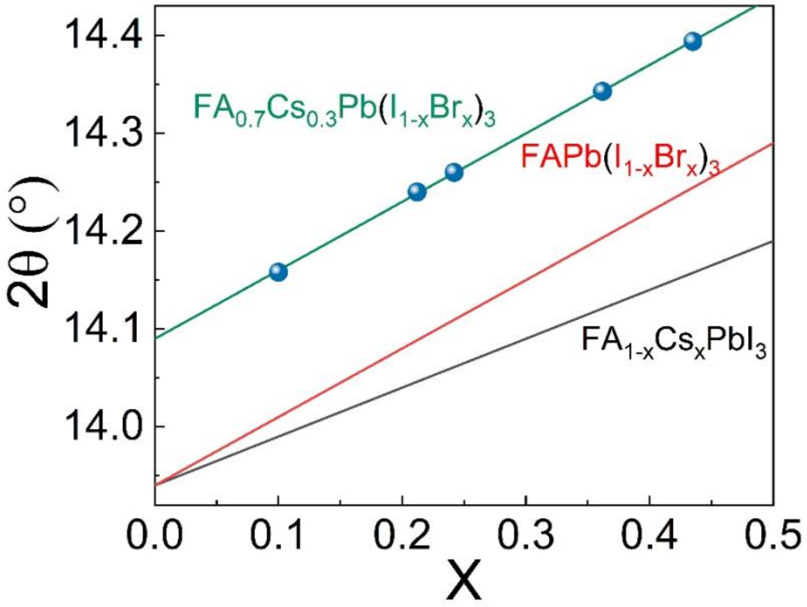

Figure S8. The evolution of lattice constant versus perovskite composition of $FA_{1-x}Cs_xPbI_3$ (black), $FAPb(I_{1-x}Br_x)_3$ (red) and $FA_{0.7}Cs_{0.3}Pb(I_{1-x}Br_x)_3$ (green). The lines are extracted from our previous publication where we prepared a series of perovskite composition based on solution-process. We measured these samples of $FA_{0.9}Cs_{0.1}Pb(I_{0.9}Br_{0.1})_3$, $FA_{0.8}Cs_{0.2}Pb(I_{0.8}Br_{0.2})_3$, $FA_{0.7}Cs_{0.3}Pb(I_{0.7}Br_{0.3})_3$ and $FA_{0.54}Cs_{0.46}(I_{0.7}Br_{0.3})_3$ with XRD and deconvolute the effect of A or X site on lattice constant shift.[2] The black line, red line and green line represent the 2θ shift based on the effect of Cs, Br and Cs/Br incorporated in perovskite structure. The blue dots are the lattice constant of evaporated perovskite films in this work with different $PbBr_2$ to PbI_2 ratio. With this trend, we assign the evaporated perovskite composition.

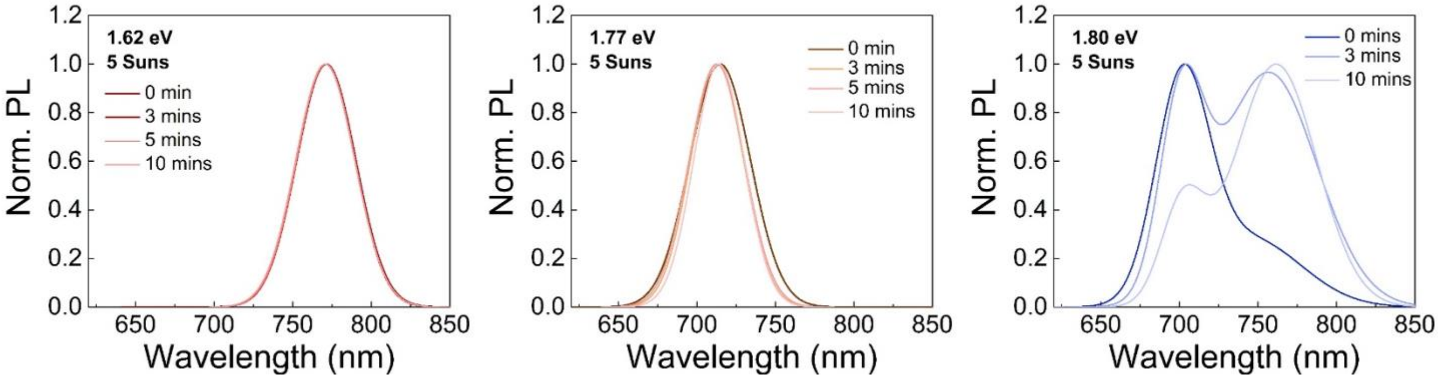

Figure S9. PL spectrum of evaporated perovskite films with different bandgap during light soaking.

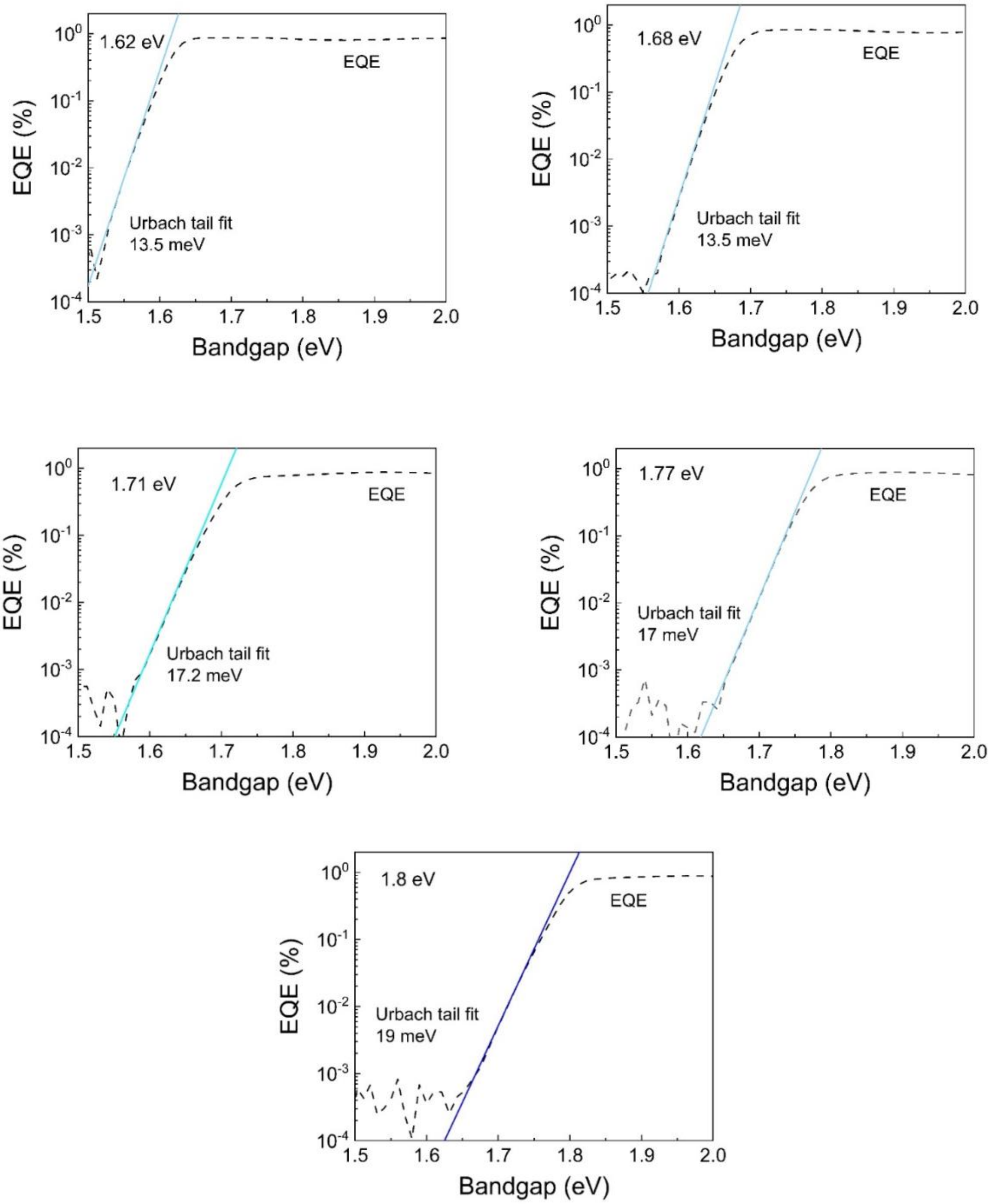

Figure S10. The Urbach tail fitting of EQE spectra of different bandgap evaporated perovskite solar cells.

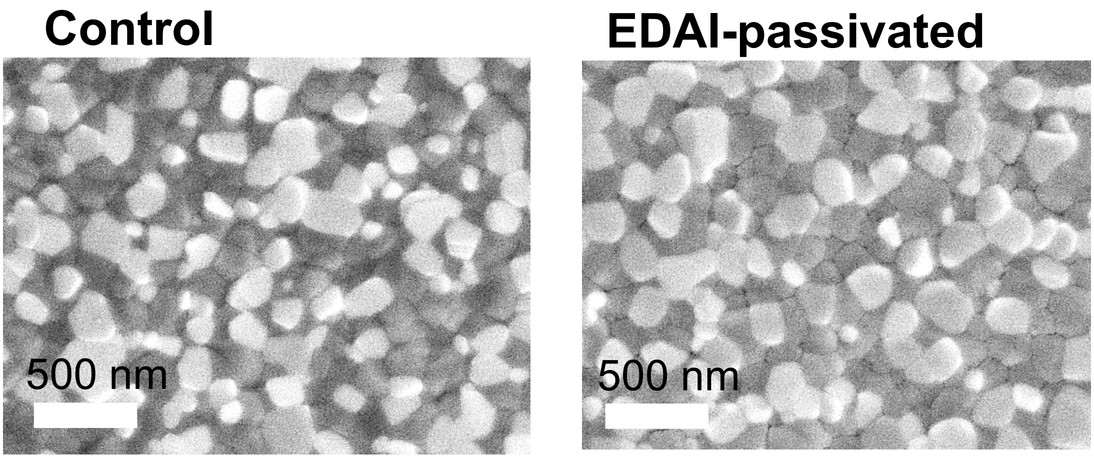

Figure S11. SEM image of 1.77 eV bandgap evaporated perovskite with $FA_{0.7}Cs_{0.3}Pb(I_{0.64}Br_{0.36})_3$composition before and after $EDAI_2$ passivation

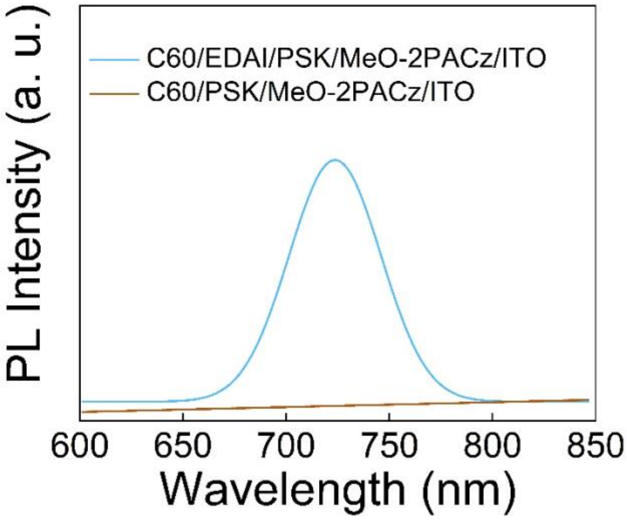

Figure S12. PL spectra of full stack evaporated perovskite $FA_{0.7}Cs_{0.3}Pb(I_{0.64}Br_{0.36})_3$ with and without $EDAI_2$ passivation.

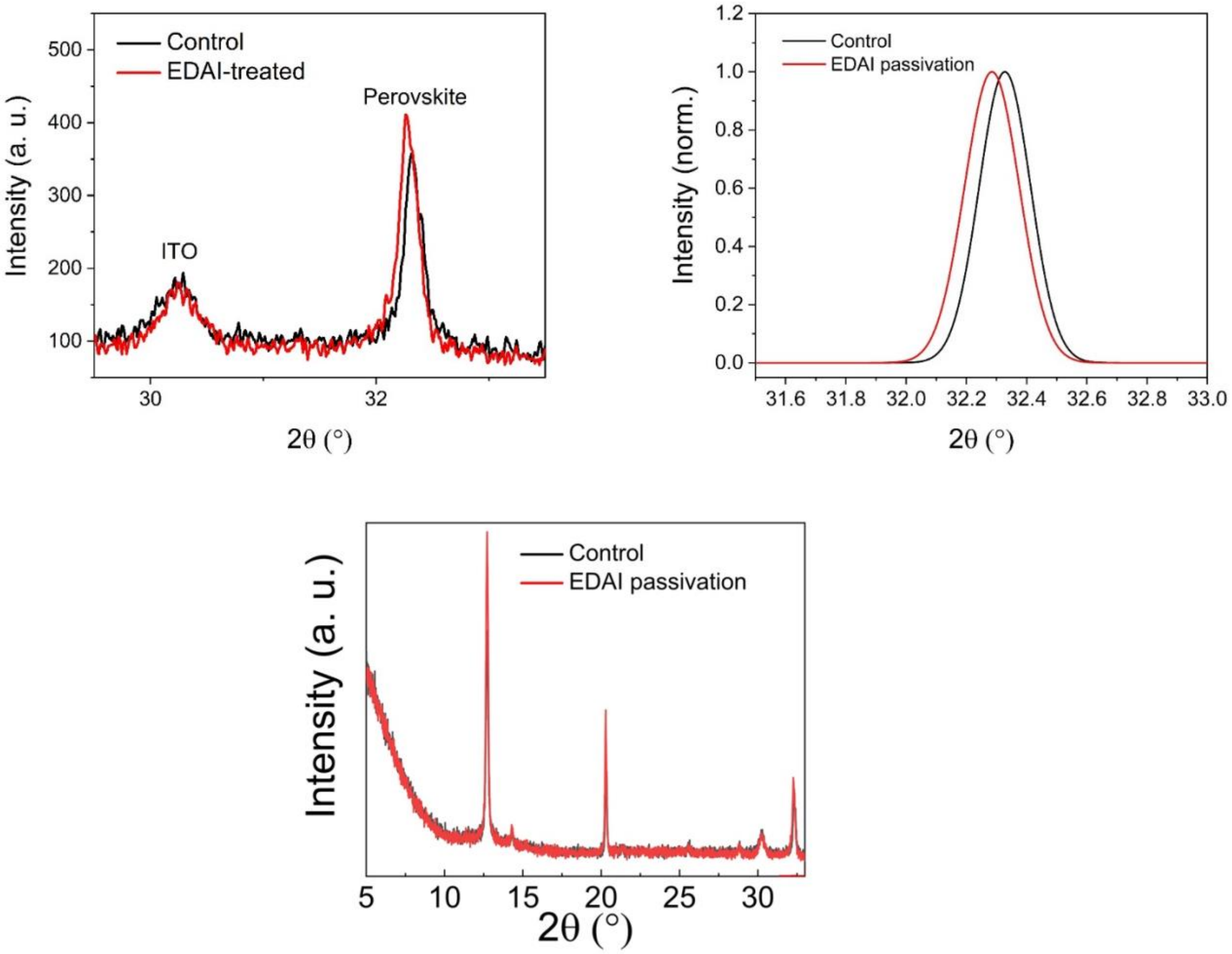

Figure S13. XRD of 1.77 eV evaporated perovskite film before and after $EDAI_2$ passivation. The ITO substrate peak is shown as a reference peak. Bottom panel shows data at lower angles discarding formation of 2D phases.

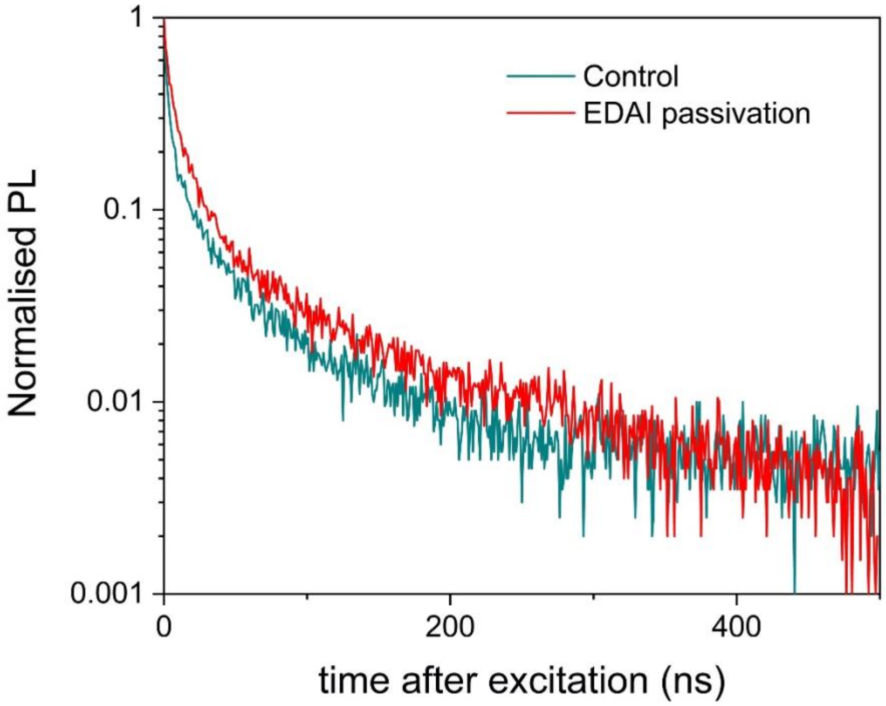

Figure S14. TRPL of evaporated perovskite films ($FA_{0.7}Cs_{0.3}Pb(I_{0.64}Br_{0.36})_3$) with and without $EDAI_2$ passivation on MeO-2PACz/ITO glass. The measurement was conducted with a 448.4 nm pulsed laser and an excited density of of 8.5 nJ/cm2/pulse,

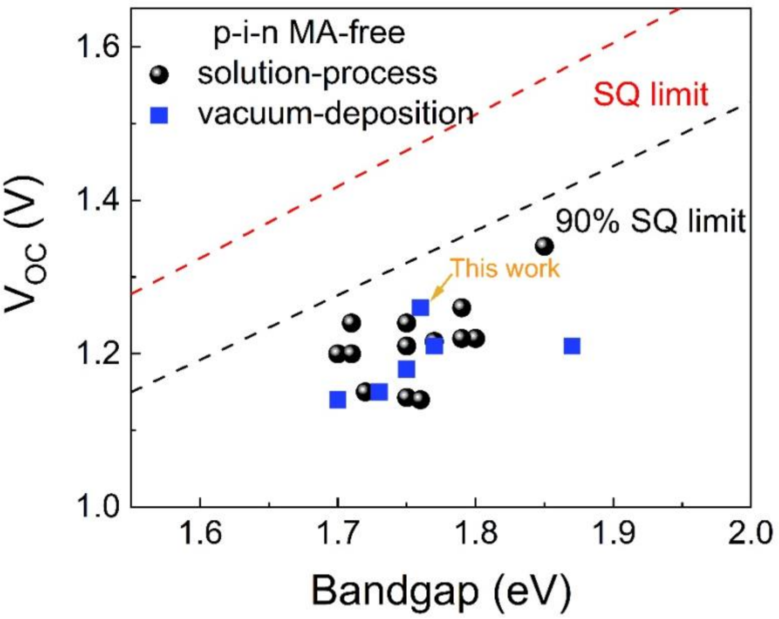

Figure S15. Literature survey of V_{OC} of solution-process or vacuum deposited widegap (>1.7 eV) film perovskite solar cells with MA-free composition. See reference 5 to 17 for solution-processed and 18, 19 for vacuum-deposited perovskite solar cells.

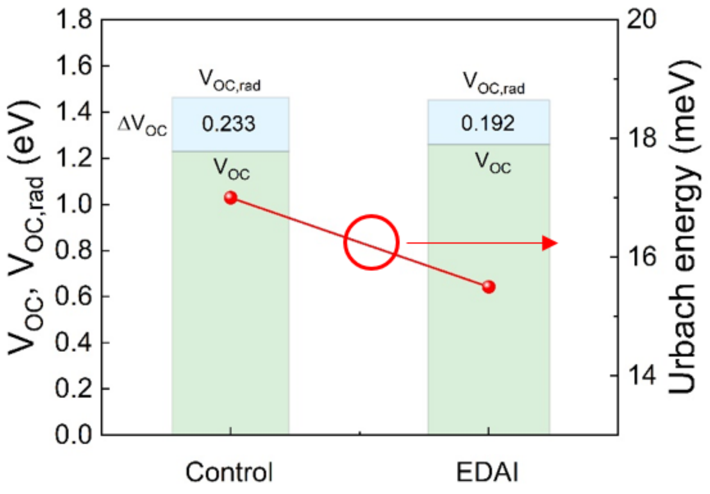

Figure S16. The measured V_{OC}, estimated $V_{OC,rad}$ and Urbach energy of wide gap perovskite solar cells ($(FA_{0.7}Cs_{0.3}Pb(I_{0.64}Br_{0.36})_3)$) with and without $EDAI_2$ passivation. The left axis is showing the V_{OC} and $V_{OC,rad}$ and the right axis is showing the Urbach energy.

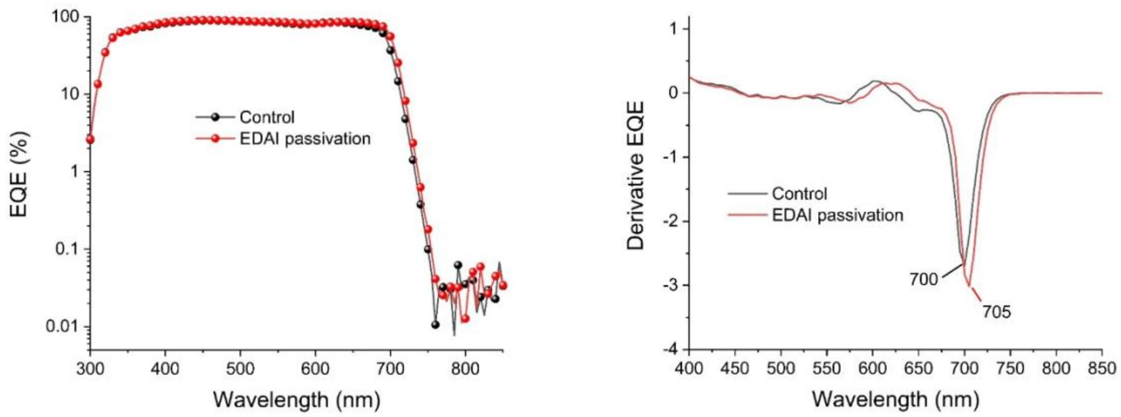

Figure S17. EQE of 1.77/1.76 eV perovskite solar cells with and without $EDAI_2$ passivation.

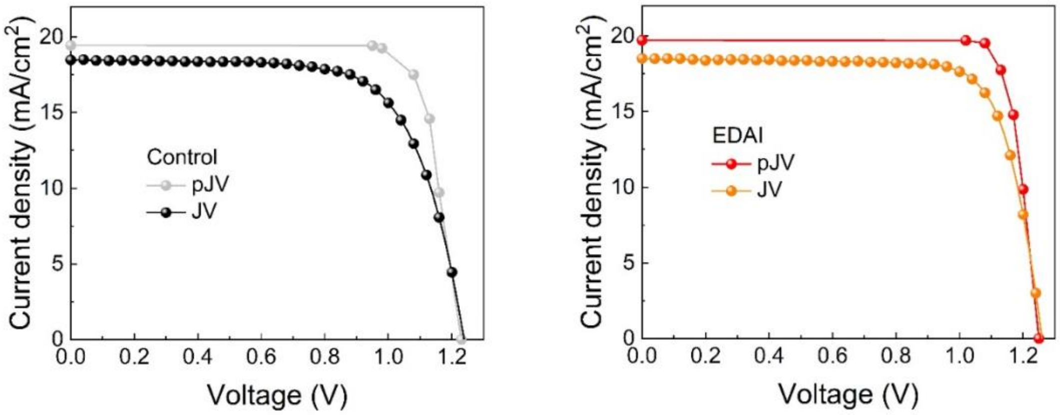

Figure S18. The pseudo-JV curve measurement of control and EDAI$_2$-treated ($FA_{0.7}Cs_{0.3}Pb(I_{0.64}Br_{0.36})_3$) perovskite solar cells. In here, we set the current density value to 95% of the radiative limit at 1 sun illumination.

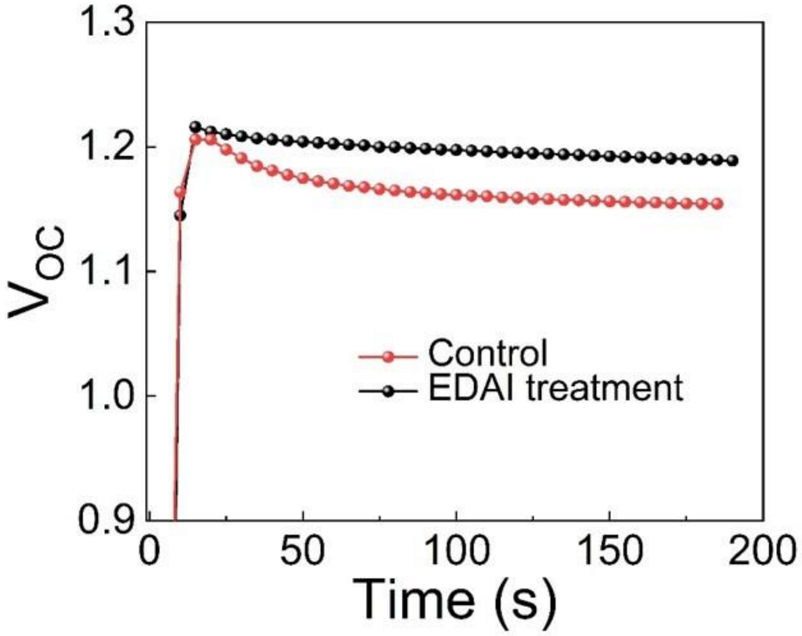

Figure S19. Voc as a function of time under AM 1.5 G illumination for control and EDAI$_2$-treated ($FA_{0.7}Cs_{0.3}Pb(I_{0.64}Br_{0.36})_3$) perovskite solar cells.

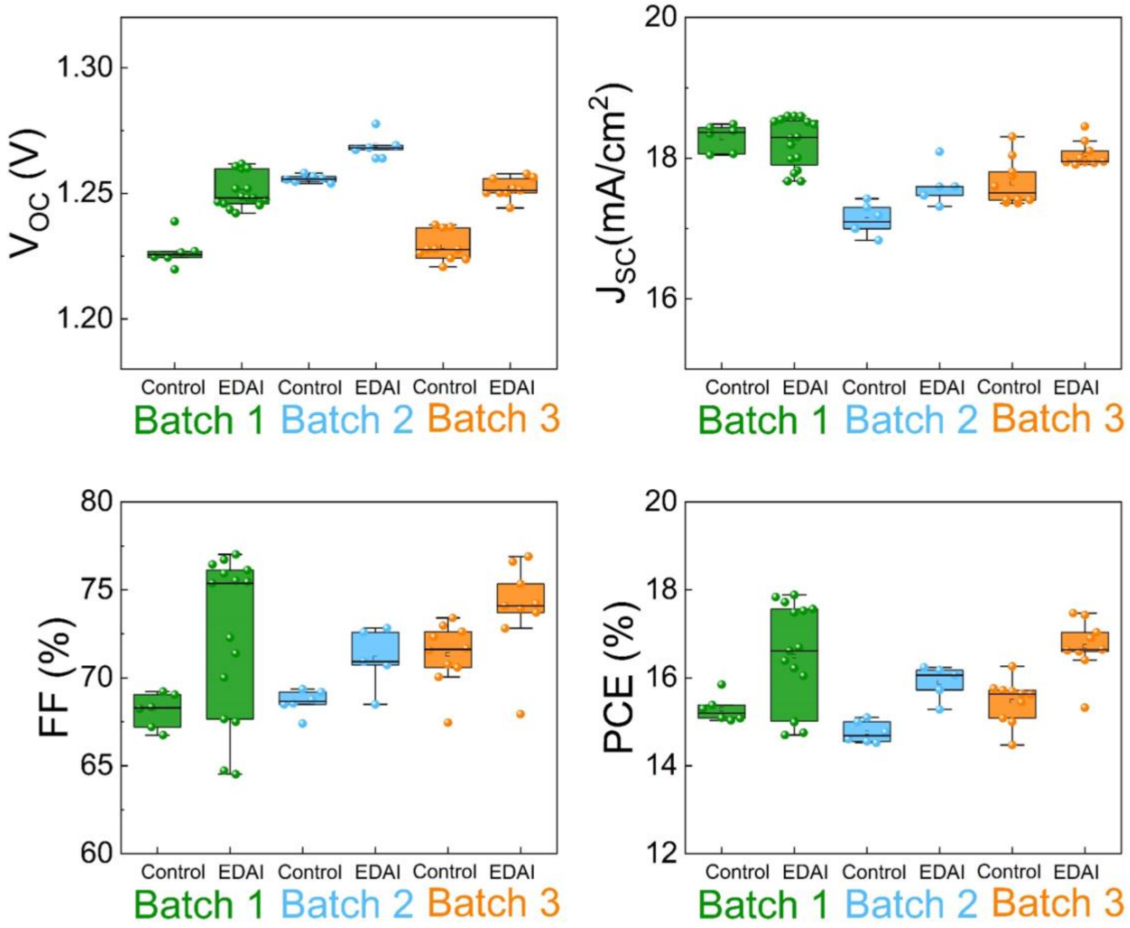

Figure S20. The batch to batch variation of V_{OC}, Jsc, FF and PCE with and without $EDAI_2$ passivation of evaporated perovskite solar cell with 1.77 eV ($FA_{0.7}Cs_{0.3}Pb(I_{0.64}Br_{0.36})_3$).

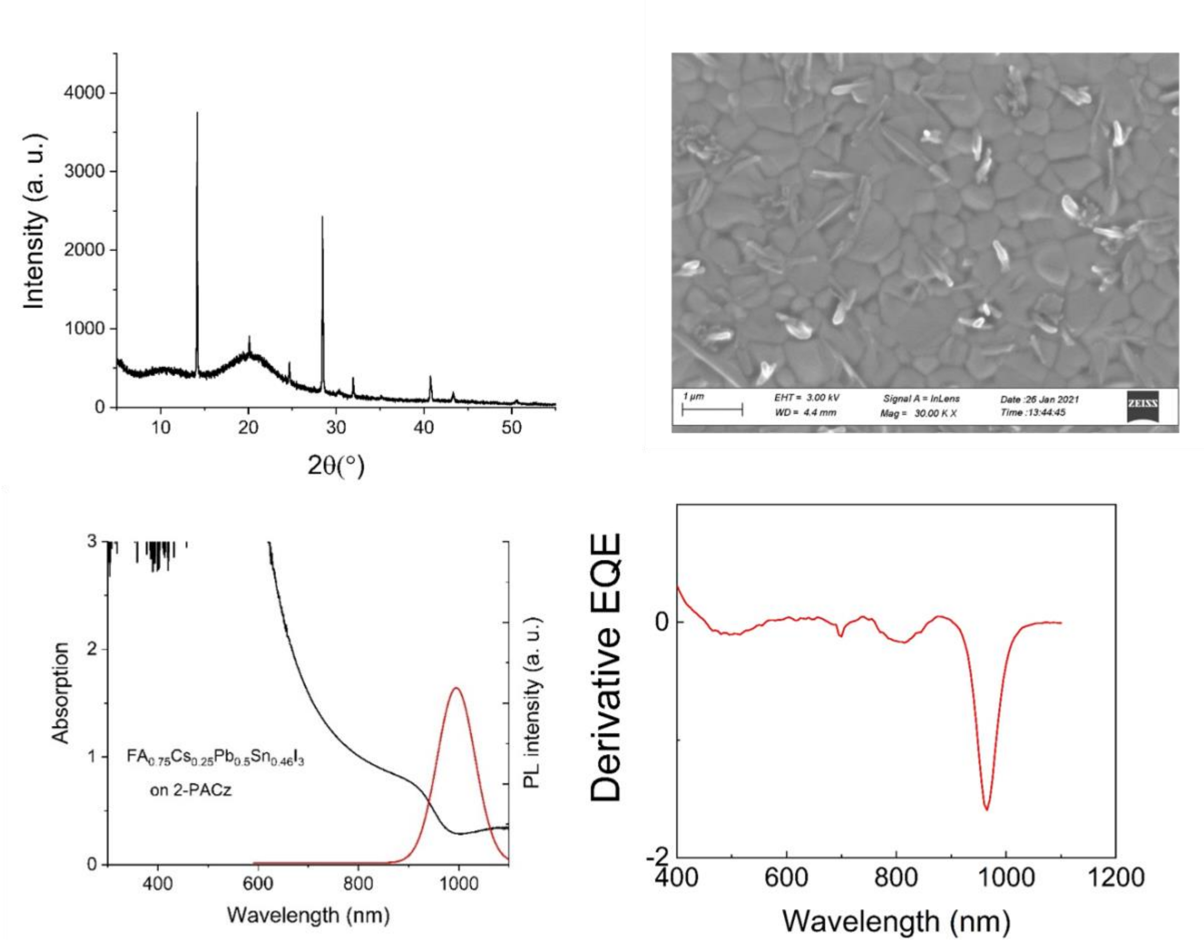

Figure S21, XRD, SEM, UV- Vis, PL and derivative EQE of narrow bandgap perovskite with composition of $FA_{0.75}Cs_{0.25}Pb_{0.5}Sn_{0.5}I_3$.

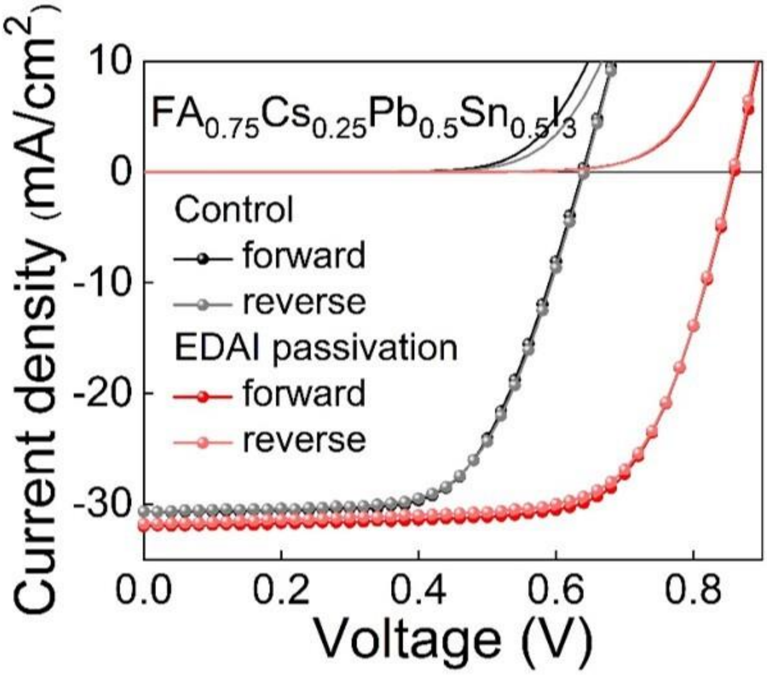

Figure S22. J-V curves for control and $EDAI_2$-passivated, $FA_{0.75}Cs_{0.25}Pb_{0.5}Sn_{0.5}I_3$ solar cell in the dark (no symbols) and under AM 1.5 G illumination (line and symbols). See device parameters in Table 2 of the main manuscript.

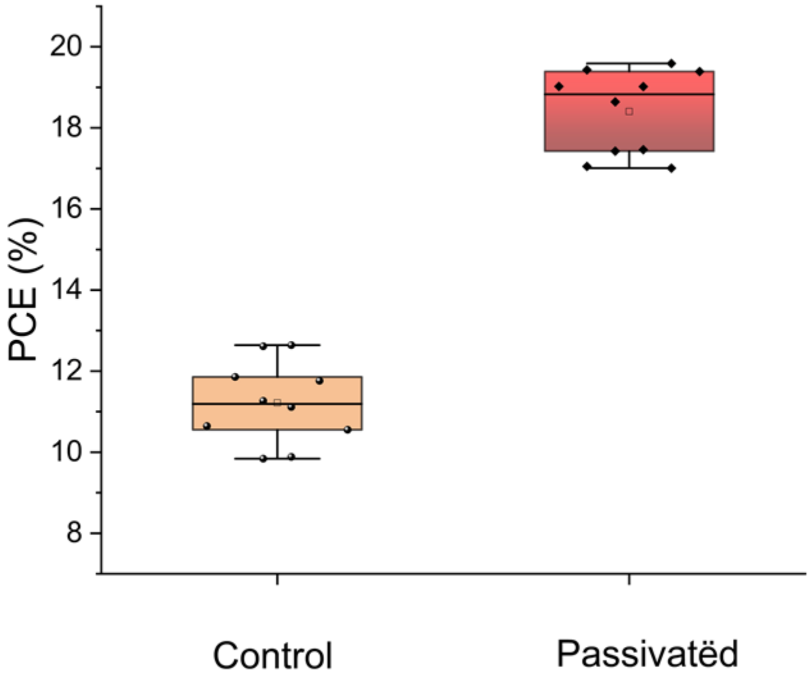

Figure S23. PCE distribution of solution-processed Pb/Sn perovskite before and after $EDAI_2$ passivation.

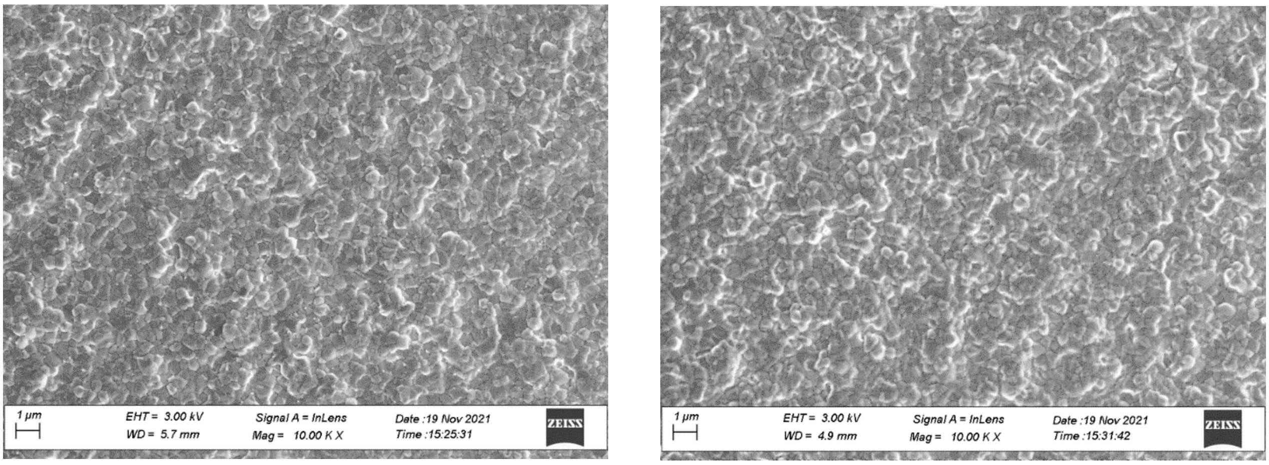

Figure S24. SEM morphology of solution-processed Pb/Sn perovskite film without (left) and with (right) $EDAI_2$ passivation.

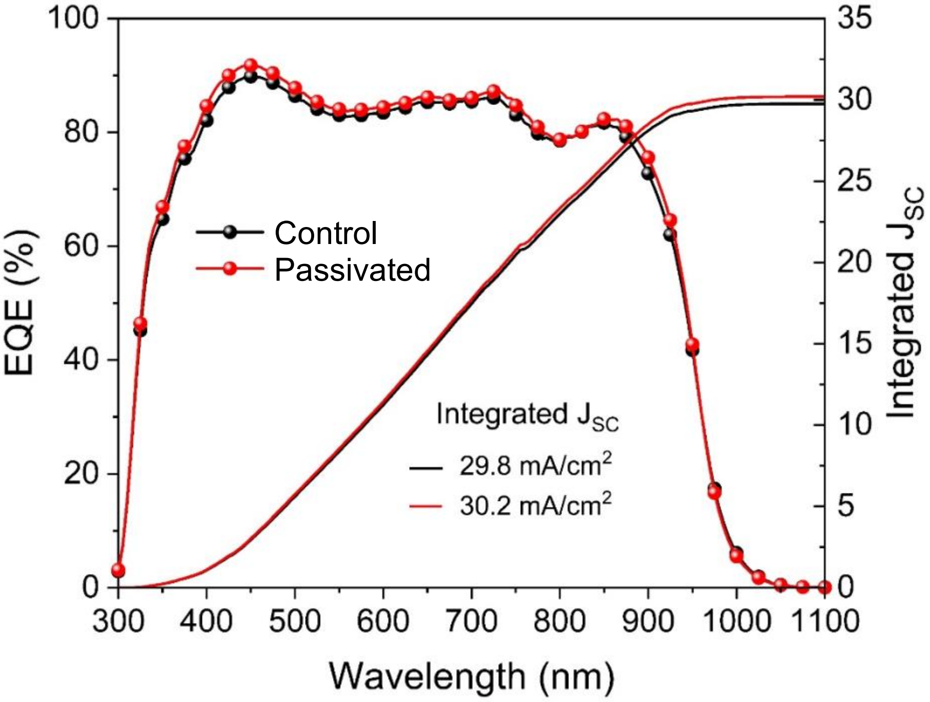

Figure S25. EQE of solution-processed Pb/Sn perovskite solar cells without (black) and with (red) $EDAI_2$ passivation.

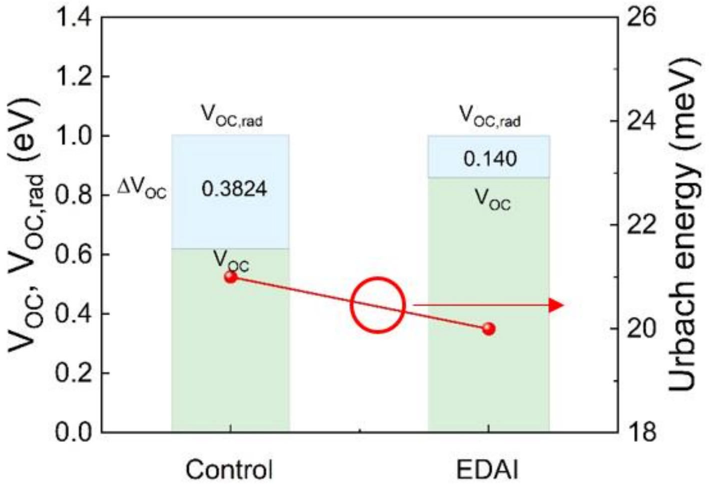

Figure S26. The measured V_{OC}, estimated V_{OC} and Urbach energy of solution-processed low gap perovskite solar cells ($FA_{0.75}Cs_{0.25}Pb_{0.5}Sn_{0.5}I_3$) with and without $EDAI_2$ passivation. The left axis shows the V_{OC} and $V_{OC,rad}$ and the right axis shows the Urbach energy.

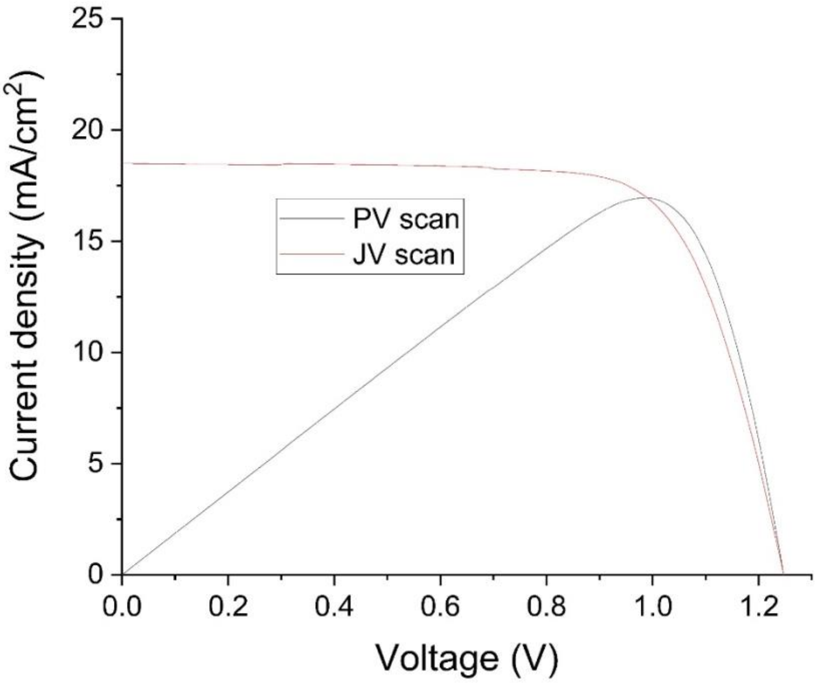

Figure S27. The J-V curve of passivated wide bandgap perovskite solar cell with 1.76 eV with ALD-SnO_x on top of C_{60} instead of BCP.

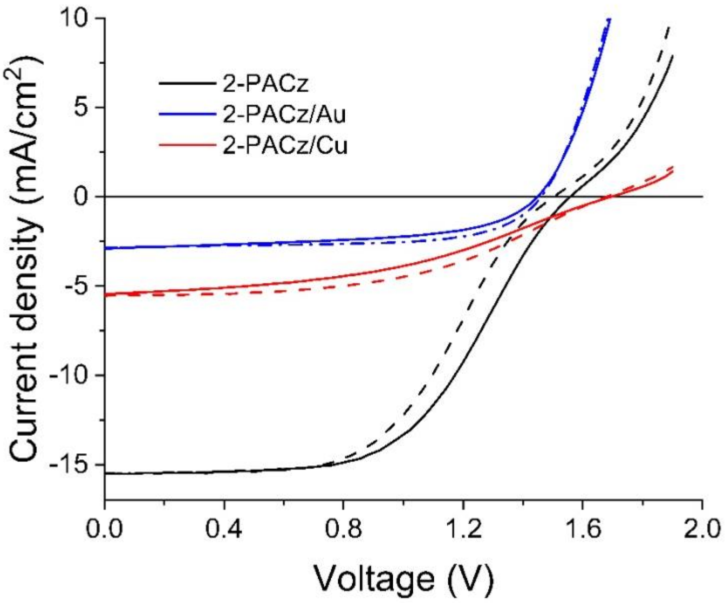

Figure S28. All perovskite tandem solar cells with different interlayers.

Table S2. The PV statistics of all-perovskite tandem solar cells with different interlayer combination.

		V_{OC} (V)	J_{SC} (mA/cm^2)	FF (%)	PCE (%)
2PACz	rev	1.56	-15.51	55.26	13.34
	for	1.50	-15.49	53.44	12.43
2PACz /Au	rev	1.45	-2.90	54.06	2.27
	for	1.46	-2.85	64.40	2.68
2PACz /Cu	rev	1.69	-5.44	42.13	3.88
	for	1.68	-5.53	48.68	4.52

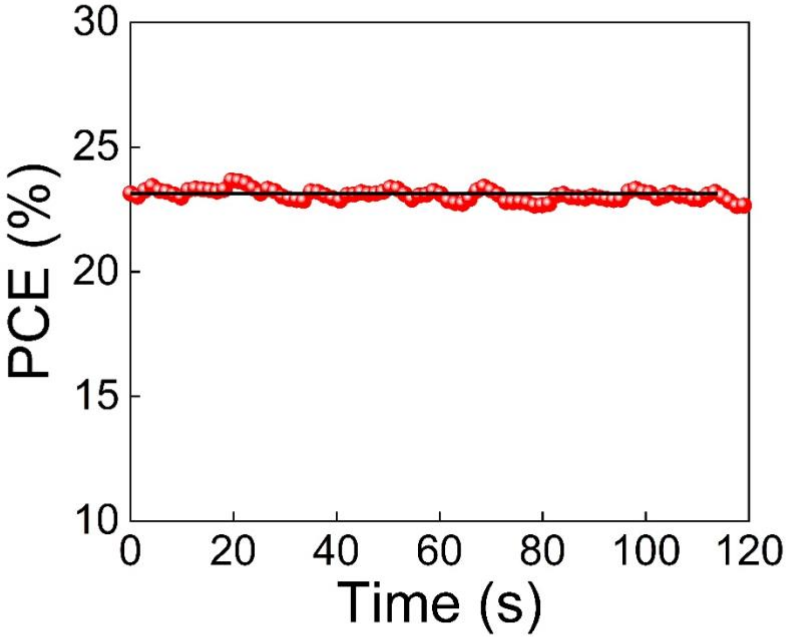

Figure S29. The stabilised power output measurement of our device with a fixed bias at 1.74 V, reaching the PCE of 23.2%.

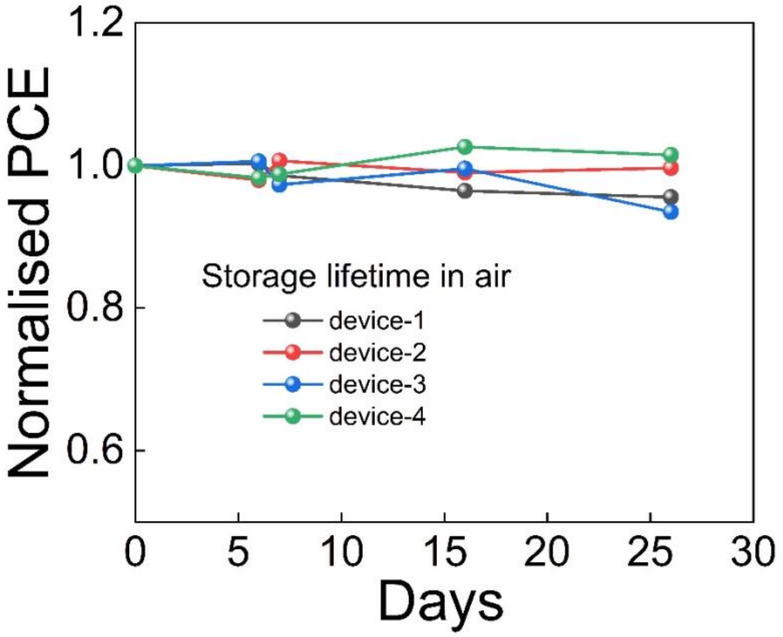

Figure S30. The storage solar cells stability test in air of encapsulated (in N_2-filled glovebox) tandem device.

Supporting Information References

1. Krückemeier, et. al. How to Report Record Open-Circuit Voltages in Lead-Halide Perovskite Solar Cells. *Adv. Energy Mater*. **2020**, *10*, 1902573.
2. Chiang, et. al. Multisource Vacuum Deposition of Methylammonium-Free Perovskite Solar Cells. *ACS Energy Lett.* **2020**, *5*, 2498–2504.
3. Mello, et. al. An Improved Experimental Determination of External Photoluminescence Quantum Efficiency *Adv. Mater.*, **1997**, *9*, 230–232.
4. Peña et al. hyperspy/hyperspy: Hyperspy v.1.5.2 (Zenodo, 2019); https://doi.org/10.5281/zenodo.3396791. Accessed December 1, 2019.
5. Palmstrom et. al. Enabling Flexible All-Perovskite Tandem Solar Cells. *Joule*, **2019**, *3*, 2193-2204.
6. Lin. et al. Matching Charge Extraction Contact for Wide-Bandgap Perovskite Solar Cells. *Adv. Mater*. **2017**, *29*, 1700607
7. Khadka et al. Tailoring the Open-Circuit Voltage Deficit of Wide-Band-Gap Perovskite Solar Cells Using Alkyl Chain-Substituted Fullerene Derivatives. *ACS Appl. Mater. Interfaces* **2018**, *10*, 26, 22074–22082.
8. Zheng. et al. Defect passivation in hybrid perovskite solar cells using quaternary ammonium halide anions and cations. *Nat Energy* **2017**, *2*, 17102.
9. Zhao, et al. Efficient two-terminal all-perovskite tandem solar cells enabled by high-quality low-bandgap absorber layers. *Nat Energy*, **2018**, *3*, 1093–1100.
10. Hu. Stabilized Wide Bandgap $MAPbBr_xI_{3-x}$ Perovskite by Enhanced Grain Size and Improved Crystallinity. **2016**, *Adv. Sci.*, *3*, 1500301.
11. Chen. Achieving a high open-circuit voltage in inverted wide-bandgap perovskite solar cells with a graded perovskite homojunction. *Nano. Energy*, **2019**, *61*, 141-147.
12. Leijtens.et al. Tin–lead halide perovskites with improved thermal and air stability for efficient all-perovskite tandem solar cells. *Sustainable Energy Fuels*, **2018**, *2*, 2450-2459.
13. Lin. et. al. Monolithic all-perovskite tandem solar cells with 24.8% efficiency exploiting comproportionation to suppress Sn(II) oxidation in precursor ink. *Nat Energy*, **2019**, *4*, 864–873.
14. Yang et. al. Enhancing electron diffusion length in narrow-bandgap perovskites for efficient monolithic perovskite tandem solar cells. *Nature Communications*, **2019**, *10*, 4498.
15. Oliver et. al. Understanding and suppressing non-radiative losses in methylammonium-free wide-bandgap perovskite solar cells. *Energy Environ.* Sci., **2022**, *15*, 714-726.
16. Brinkmann. et. al. Perovskite–organic tandem solar cells with indium oxide interconnect. *Nature*, **2022**, *604*, 280–286.
17. Chen. et. al. Monolithic perovskite/organic tandem solar cells with 23.6% efficiency enabled by reduced voltage losses and optimized interconnecting layer. *Nat Energy*, **2022**, *7*, 229–237.
18. Gil-Escrig. et al. Efficient Wide-Bandgap Mixed-Cation and Mixed-Halide Perovskite Solar Cells by Vacuum Deposition. *ACS Energy Lett*. **2021**, *6*, 827–836.
19. Longo. et al. Fully Vacuum-Processed Wide Band Gap Mixed-Halide Perovskite Solar Cells. *ACS Energy Lett*. **2018**, *3*, 214–219.
